# A fully automated noncontrast CT 3‐D reconstruction algorithm enabled accurate anatomical demonstration for lung segmentectomy

**DOI:** 10.1111/1759-7714.14322

**Published:** 2022-02-09

**Authors:** Xiuyuan Chen, Zhenfan Wang, Qingyi Qi, Kai Zhang, Xizhao Sui, Xun Wang, Wenhan Weng, Shaodong Wang, Heng Zhao, Chao Sun, Dawei Wang, Huajie Zhang, Enyou Liu, Tong Zou, Nan Hong, Fan Yang

**Affiliations:** ^1^ Department of Thoracic Surgery Peking University People's Hospital Beijing China; ^2^ Department of Radiology Peking University People's Hospital Beijing China; ^3^ Institute of Advanced Research Infervision Medical Technology Co., Ltd Beijing China

**Keywords:** 3D reconstruction, artificial intelligence, lung, noncontrast CT, segmentectomy

## Abstract

**Background:**

Three‐dimensional reconstruction of chest computerized tomography (CT) excels in intuitively demonstrating anatomical patterns for pulmonary segmentectomy. However, current methods are labor‐intensive and rely on contrast CT. We hereby present a novel fully automated reconstruction algorithm based on noncontrast CT and assess its performance both independently and in combination with surgeons.

**Methods:**

A retrospective pilot study was performed. Patients between May 2020 to August 2020 who underwent segmentectomy in our single institution were enrolled. Noncontrast CTs were used for reconstruction. In the first part of the study, the accuracy of the demonstration of anatomical variants by either automated or manual reconstruction algorithm were compared to surgical observation, respectively. In the second part of the study, we tested the accuracy of the identification of anatomical variants by four independent attendees who reviewed 3‐D reconstruction in combination with CT scans.

**Results:**

A total of 20 cases were enrolled in this study. All segments were represented in this study with two left S1‐3, two left S4 + 5, one left S6, five left basal segmentectomies, one right S1, three right S2, 1 right S2b + 3a, one right S3, two right S6 and two right basal segmentectomies. The median time consumption for the automated reconstruction was 280 (205–324) s. Accurate vessel and bronchial detection were achieved in 85% by the AI approach and 80% by Mimics, *p* = 1.00. The accuracy of vessel classification was 80 and 95% by AI and manual approaches, respectively, *p* = 0.34. In real‐world application, the accuracy of the identification of anatomical variant by thoracic surgeons was 85% by AI+CT, and the median time consumption was 2 (1–3) min.

**Conclusions:**

The AI reconstruction algorithm overcame defects of traditional methods and is valuable in surgical planning for segmentectomy. With the AI reconstruction, surgeons may achieve high identification accuracy of anatomical patterns in a short time frame.

## Graphical abstract

Intraoperative observation of three example cases (Ac, Bc, Cc) and the 3D reconstruction by manual (Ab, Bb, Cb) and automated (Aa, Ba, Ca) approach. All structures were correctly reconstructed in Aa and Ba. The interlobular vein failed to be reconstructed in the automated reconstruction (Ba) and manual reconstruction (Bb). V7a was wrongly recognized as A7a in the automated reconstruction (Ca).

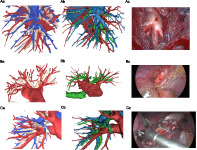

## INTRODUCTION

Lung cancer is the leading cause of cancer‐related death worldwide, with an estimated 1.8 million deaths in 2021 according to the GLOBOCAN estimation.^1^ Thanks to the increasing application of computerized tomography (CT), the detection rate of early stage lung cancer, especially preinvasive lesions such as ground‐glass nodules (GGN), is rapidly increasing.^2^ Accumulating data from studies have indicated that sublobar resection, especially anatomic segmentectomy, is an optimal treatment for these lesions.^3^, ^4^

A major challenge of thoracoscopic segmentectomy to surgeons is that they should be cautious and thorough familiar with anatomical variants in pulmonary vessels and bronchi, for the anatomy is barely standard.^5^ False recognition of anatomical variants in pulmonary vessels, such as mediastinum A^4+5^ or extra subdivisions of V^2^t, might result in massive bleeding during surgery. Chest CT imaging plays an essential role in the pulmonary surgery planning process, especially for intricate procedures such as segmentectomy. However, mastering chest CT at a segmental or subsegmental level is challenging due to its less intuitive representation. Even for experienced surgeons, misdetection and misclassification of remote PAs and PVs may still occur and potentially cause bleeding, mis‐ligation, or other catastrophic consequences. Compared to traditional 2D CT images, 3D reconstruction is more intuitive in illustrating 3‐dimensional variants of vessels and bronchi. Thus, a reliable reconstruction method on the basis of CT images may be highly beneficial for surgeons during operative planning.^6^, ^7^

Researchers have attempted to use handcraft or semi‐automatic tools, such as “Mimics” or “Visible Patient” etc., for three‐dimensional computed tomography bronchography and angiography (3D‐CTBA). These methods are being experimentally used in the clinic with the following defects. First, delimitation of each segment in the lobe of interest in traditional post‐processing methods based on HU level, which enable software to distinguish vessels from parenchyma but not from infection or tumor. The identification accuracy of segmental pulmonary arteries has previously been reported to range from 62% to 90% (mainly on contrast CT).^8^, ^9^ Second, manual reconstruction is time‐consuming, and the processing time can take up to 1 h for radiological technicians.^10^ Moreover, manual reconstruction relies mostly on contrast CTs, while it is not always necessary for patients intended to undergo segmentectomy to receive preoperative contrast CT, especially for patients with pure or mixed GGN.

On the contrary, deep learning based automatic segmentation technology has contributed to improved accuracy and time efficiency on radiological image segmentation in a laboratory setting;^11^ however, the clinical evidence for applying such approach is still in high demand.

In this study, we assessed the performance of an automatic imaging reconstruction system, InferVisual Surgery Planning (Research version), in assisting thoracic surgeons in preoperative planning. This system was evaluated retrospectively in efficiency, accuracy and robustness using 20 CTs from patients who underwent segmentectomy. Here, we present the following article in accordance with the STROBE reporting checklist.

## METHODS

### Definition of accuracy

The gold standard for the types of pulmonary structures and patterns of anatomical variants were established based on intraoperative findings in combination with CT scans. For the independent performance test, the accuracy of detection was defined as successful detection of targeted pulmonary structures in the 3‐D reconstruction divided by total related structures, and the accuracy of vessel classification was defined as correct discrimination between arteries and veins divided by total number of related PAs and PVs. The overall accuracy was defined as number of successful detected and correctly classified structures divided by the number of all related structures. For the surgeon‐AI combined test, a nonexhaustive form of anatomical variants (Table S1) was created according to Hiroaki's publication,^12^ clinical observation and other articles.^13^, ^14^, ^15^ Each column of the form represents a set of common anatomical variants regarding one segmental structure (PAs, PVs or bronchi). Accuracy was defined as the number of correctly selected structures divided by the number of all related structures during operation.

### Patient enrollment

Patients who underwent segmentectomy at Peking University People's Hospital between May 2020 to August 2020 were retrospectively reviewed. The inclusion criteria were as follows: (1) preoperative thin‐section (<1.25 mm), noncontrast CT images available at our institution, and (2) time from CT examination to surgery less than 1 month. Among patients who met the above criteria, we arbitrarily selected 20 cases representing most of the common segmentectomies.

Chest CT scans were assessed for image quality by a thoracic radiologist. A scoring system (Table S2) consisting of a number of quality criteria was utilized to measure the CT image quality. Dicom information is shown in Table S3.

### DL‐based surgery planning assistance system

**FIGURE 1:**
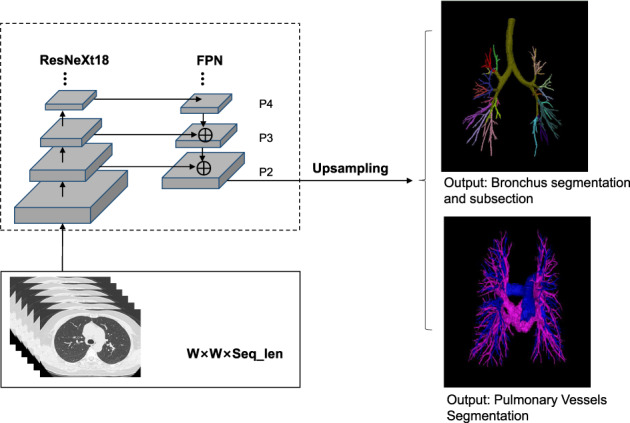
Schematic roadmap of DL‐based surgery planning assistance system

In this study, a surgery planning assistance system developed using deep learning technology (InferVisual Surgery Planning Research version) was utilized and validated in terms of its auxiliary role in segmentectomy surgery planning. Fine annotated CT scans in which the pulmonary blood vessels and bronchi were concisely segmented to subsegment level by senior thoracic surgeons were used for modeling; deep learning region segmentation and region growth modules were jointly utilized in the models development; a schematic roadmap is shown in Figure 1. The DL‐based system can automatically complete and display the 3D reconstruction of pulmonary blood vessels and bronchi based on either enhanced CT scans or plain CT scans, thereby helping thoracic surgeons to plan for lobectomy and segmentectomy. The accessibility to noncontrast CT scan can significantly reduce the radiation dose exposed to the patient.

### Study variables and statistical analysis

Study variables included patient demographics and clinical features (age, sex, smoking history, lung function, CT‐index), tumor characteristics (tumor location, tumor size, histology), surgery characteristics (blood loss, operation time). In the part of independent performance assessment, the accuracy of the demonstration of anatomical variants by either automated (by InferVisual Surgery Planning Research version) or manual reconstruction algorithm (by Mimics software) were calculated based on the comparison with surgical observation. In the second part of the surgeon‐AI combined performance assessment, the accuracy of identification of anatomical variants by four independent attendees who reviewed 3‐D reconstruction in combination with CT scans was calculated based on the same gold standard. Continuous data were analyzed using the Mann–Whitney test and the categorical variables were processed by Chi square or Fisher's exact tests, as appropriate.

## RESULTS

### Clinical characteristics

**FIGURE 2:**
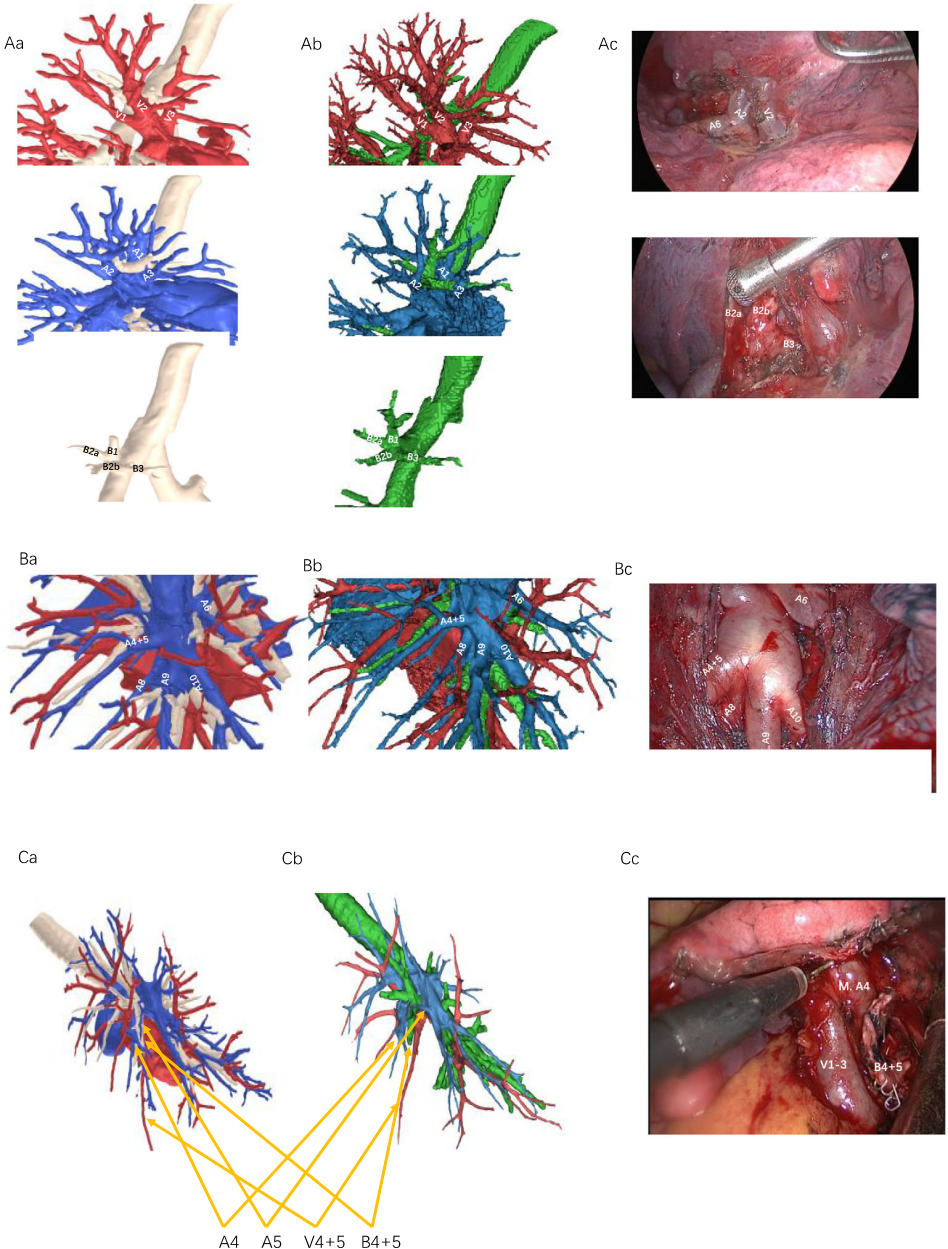
Intraoperative observation of three example cases (Ac, Bc, Cc) and the 3D reconstruction by the manual (Ab, Bb, Cb) and AI (Aa, Ba, Ca) approach

A total of 77 segmentectomies were performed in our institution from May 2020 to August 2020. All dicom files are available. We arbitrarily selected 20 cases representing most of the common segmentectomies, which included two left S1–3, two left S4 + 5, one left S6, five left basal segmentectomies, one right S1, three right S2, one right S2b + 3a, one right S3, two right S6 and two right basal segmentectomies. The clinical characteristics are delineated in Table 1. The median radiological index was 3 (2.75–4). All enrolled cases were successfully reconstructed by both AI algorithm and manual approach (Figure 2, Figure S1), and no systemic failures were observed. Manual reconstruction was carried out in a 30 min timeframe, while the median time of AI reconstruction was 280 (205–324) s.

**TABLE 1:** Patient and surgical characteristics

Variable	All	Without error	With error	*p*‐value	
Number of cases, *n*	20	13	7	
Age, median (IQR), year	58 (50.8–62.5)	58 (49–59)	62 (53–63)	0.42
Sex, *n* (%)				0.92	
Female	14 (70.0)	9 (69.2)	5 (71.4)		
Male	6 (30.0)	4 (30.8)	2 (28.6)		
Smoking history, *n* (%)	4 (20.0)	3 (23.1)	1 (14.3)	0.64
FEV1/FVC, mean (IQR), %	79.98 (75.83–83.57)	80.5 (77.7–83.6)	78.8 (73.3–82.9)	0.54
FEV1, median (IQR), L	2.38 (2.15–2.73)	2.4 (2.2–2.7)	2.5 (2.1–2.7)	0.86
Histology, *n* (%)				0.73
Benign lesion	1 (5.0)	1 (7.7)	0 (0)	
AAH	1 (5.0)	1 (7.7)	0 (0)		
MIA	6 (30.0)	4 (30.8)	2 (28.6)		
Invasive adenocarcinoma	12 (60.0)	7 (53.6)	5 (71.4)	
Tumor location				0.37
RUL	6 (30.0)	3 (23.1)	3 (42.9)		
RLL	4 (20.0)	2 (15.4)	2 (28.6)		
LUL	4 (20.0)	4 (30.8)	0 (0)		
LLL	6 (30.0))	4 (30.8)	2 (28.6)		
Tumor size, median (IQR), cm	1.25 (1–2.75)	1.3 (1–1.5)	1.2 (1–1.5)	0.72
CT‐index, median (IQR)	3 (2.5–4)	4 (3–4)	3 (2–3)	0.20
Blood loss, median (IQR), ml	30 (20–50)	30 (20–50)	50 (20–50)	0.34
Operation time, median (IQR), minutes	167.5 (137.5–230)	165 (120–210)	170 (150–230)	0.50

### Independent performance of reconstruction approaches

The overall accuracy of all segmental and subsegmental vessels was 0.70 for the automated approach and 0.80 for the manual approach, *p* = 0.72. Accurate vessel and bronchi detection was achieved in 85% by the AI model and 80% by Mimics, *p* = 1.00. The accuracy of vessel classification was 80% and 95% by AI and manual approaches, respectively, *p* = 0.34. The AI algorithm showed four misclassifications and three misdetections, while the manual method showed four misdetections and only one misclassification (Table 2). Age, sex, smoking history, pathological type, radiological score, tumor location and tumor size were not associated with reconstruction error (Table 1).

**TABLE 2:** Independent performance analysis

Evaluation factor	AI	Mimics	*p*‐value
Overall accuracy	0.7	0.8	0.72
Detection accuracy	0.85	0.8	1.00
Classification accuracy	0.8	0.95	0.34
Risky error rate	0.15	0.15	1.00

### Error analysis of reconstruction approaches

**FIGURE 3:**
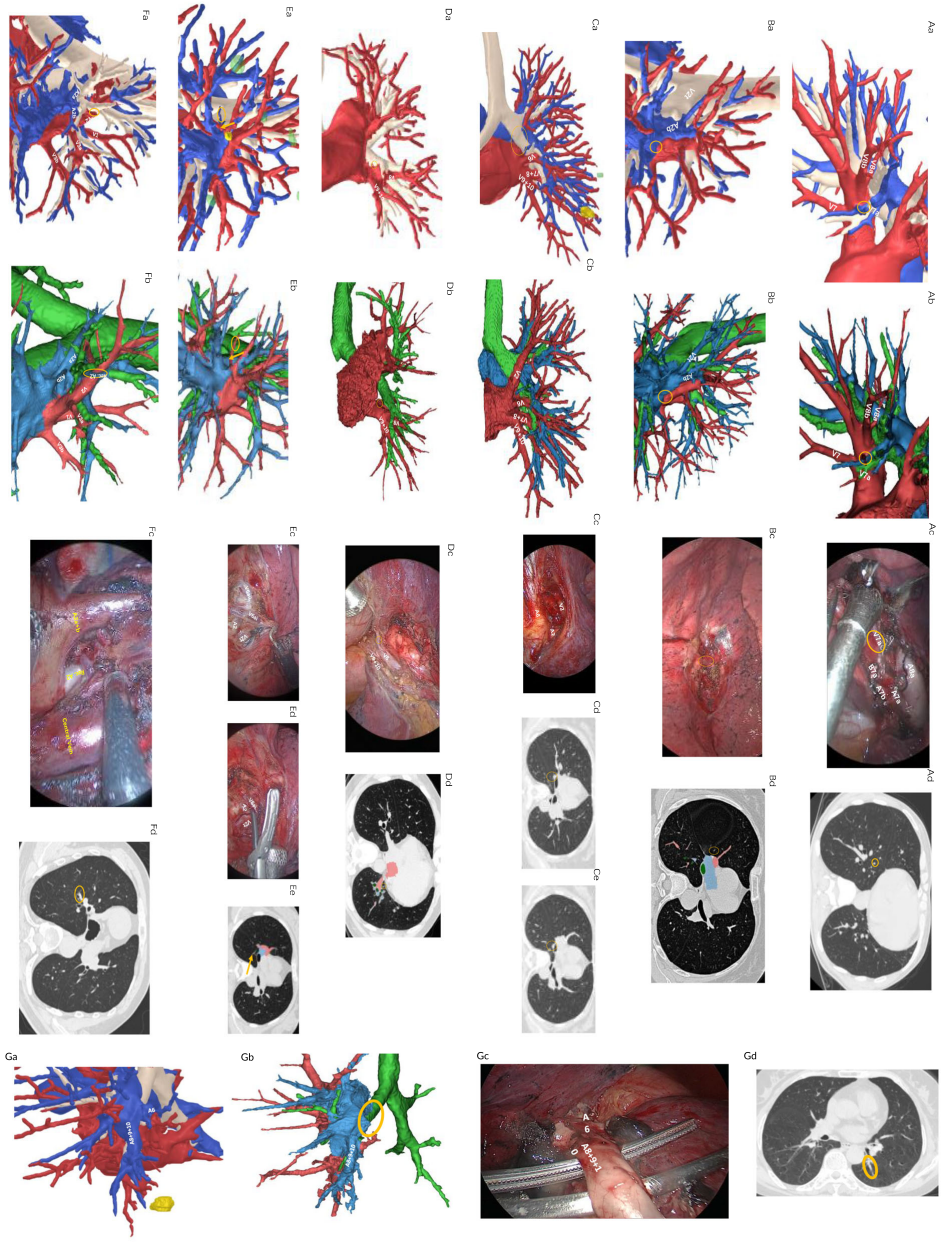
Error cases during the independent performance assessment. Patient 3 (A): misclassification in the automated reconstruction. V7a (Ac, Ad) was wrongly recognized as A7a in the automated reconstruction (Aa), which was successfully depicted in the manual reconstruction (Ab). Patient 4 (B): misclassification and misdetection in the automated and manual reconstructions. V2t was misclassified and an interlobular vein (Bc, Bd) was missed (yellow circle) in the automated reconstruction (Ba) and the manual reconstruction (Bb). Patient 5 (C): misdetection in the automated reconstruction. Proximal part of V2 (Cd, Ce, yellow circle) was absent in the automated reconstruction (Ca), which was successfully depicted in the manual reconstruction (Cb). Patient 8 (D): misdetection in the automated and manual reconstructions. The interlobular vein (Dd) identified during the operation (Dc) failed to be reconstructed in the automated reconstruction (Da) and the manual reconstruction (Db). Patient 11 (E): misclassification in the automated reconstruction and misdetection in the manual reconstruction. The V6i (yellow circle) was misclassified in the automated reconstruction (Ea), and V6aii (Ee, yellow arrow) was missed in the manual reconstruction (Eb). Patient 13 (F): misclassification in the automated reconstruction. The A3a (Fd, yellow circle) was misclassified in the automated reconstruction (Fa). Patient 15 (G): misdetection in the manual reconstruction. A6 (Gc, Gd) was absent in the manual reconstruction (Gb), which was successfully depicted in the automated reconstruction (Ga)

In AI reconstruction, misclassifications were seen in one V7a, one V^2^t, one V6i and one A3a, and misdetections were seen in two interlobular veins, one proximal part of V2. In manual reconstruction, misclassifications were seen in one V^2^t, and misdetections were seen in two interlobular veins, one V6aii and one A^6^ (Figure 3). According to the assessment of two individual attendees, three errors from AI and manual reconstruction were considered a surgical risk for they may cause ligation of the wrong vessel or potential bleeding during segmentectomy (risk error rate 15%).

### Real‐world performance assessment

To mimic the preoperative planning scenario, AI reconstructions in combination with original CT scans of enrolled cases were assessed by four individual attendees to distinguish anatomical variants of PAs, PVs and bronchi of resected segment. The average accuracy of all anatomical structures assessed was 0.85, and 0.79, 0.80, 0.96 for PAs, PVs and bronchi, respectively (Table 3, Table S4). The median time consumption for one case was 2 min.^1^, ^2^, ^3^ Clinicopathological characteristics, radiological score and type of surgery were not associated with diagnostic error.

**TABLE 3:** Accuracy of the combination of surgeon, AI and CT scan

Accuracy	All variance	PAs	PVs	Bronchi
Agent A	0.88	0.86	0.79	0.96
Agent B	0.86	0.79	0.88	0.93
Agent C	0.84	0.79	0.79	0.93
Agent D	0.84	0.75	0.75	1.00
Average	0.85	0.79	0.80	0.96

### Error analysis of AI reconstruction with original CT scans

In this part of the real‐world application study, misdiagnosis was observed in 19 segmental or subsegmental structures (Table 4). Specifically, identification of the variant of LV^6^ showed a lowest accuracy of 25.0%, and only one attendee correctly identified the type of LV^6^ with three stems (V^6a^ + V^6b^ + V^6c^). Both the preoperative diagnosis of RA^8^ and RA^3^ showed an accuracy of 50%. Half of the anatomical structures selected in this study were assessed with an accuracy of 100%.

**TABLE 4:** Accuracy of AI reconstruction with original CT scans

Anatomical structure	Accuracy rate
RUL	
A1	1.00
A2	0.94
A3	0.50
B1‐3	1.00
V1	1.00
V2	0.94
V3	0.75
RLL	
A6	0.75
A8	0.50
A9	1.00
B6	1.00
B8	1.00
B9	1.00
V6	0.75
V8	0.75
LUL	
A1 + 2	1.00
A3	0.63
A4	1.00
A5	1.00
B1 + 2	1.00
B1‐3	0.88
B3	0.63
B4	1.00
B5	1.00
V1 + 2 + 3	1.00
V4 + 5	0.75
LLL	
A6	1.00
A8	0.75
A9	0.75
A10	0.56
B6	0.75
B8	1.00
B9	1.00
B10	1.00
V6	0.25
V8	1.00
V9	0.92
V10	0.56

## DISCUSSION

In this study, we first compared the independent performance of the deep‐learning algorithm and traditional manual approach on 3‐D reconstruction of chest CT. In addition, we assessed the clinical value of this automated algorithm in surgery planning scenario by identifying anatomical variants of pulmonary arteries, veins and bronchial by four individual attendings.

In our results, the automated algorithm attained an overall accuracy of 70% in terms of independent performance, which seemed to be lower than another automated model with an accuracy of 94% reported by Nardelli et al.^11^ However, lower accuracy might not necessarily reflect less clinical value. Of note, errors in our AI reconstruction were mainly observed in distant vessels which was not our primary focus because of little impact on procedure (Table S5). The previous publication optimizes the classification of distal structures and renders outstanding classification performance of distal PAs and PVs. In the segmentation surgery planning scenario, however, proximal structure detection, especially on the segmental and lobular level, requires higher accuracy.

Our results showed that the deep learning‐based algorithm achieves similar accuracy independently comparing to manual approach. The automated algorithm showed a trend of better performance in vessels and bronchi detection while manual reconstruction was trending toward more accurate vessel classification. For example, in patient 15 (Figure S15), A^6^ was identified in automated reconstruction but misdetected in the manual approach, while in patient 3 (Figure 3), V^7^a was misclassified as A^7^a in automated approach. In fact, as we examined all automated 3D reconstructions, the approach tended to be more sensitive in identifying fine vessels regardless of whether it was distal or proximal but was less optimal in classification of distal branches, especially in adjacent distal PAs and PVs. Such characteristics are more favored by surgeons compared to the manual approach since the misdetection of small vessels may cause serious bleeding,^16^ while misclassification can usually be corrected intraoperatively before ligation. For example, the misdetection of the interlobular vein which is a subsegmental branch of V^5^ drains into V^8^ (patient 8, Figure S8) was risky for S7 + 8 segmentectomy, while the misclassification of V^6^ai as a PA by AI (patient 11, Figure S11) had little influence on the S2 segmentectomy comparing to the misdetection of the V^6^aii by manual approach. These results suggested that, despite few flaws in the classification of distant vessels, the automated approach could represent the 3‐D structure with a surgically favored high accuracy.

The time consumed in the process of reconstruction is also a key point to consider during clinical practice, and the higher accuracy generally requires much more time. It is possible that with enough caution and unlimited time, manual reconstruction may reach 100% accuracy.^17^, ^18^ However, in a clinically reasonable time frame, such accuracy cannot be reached. The median time consumption of the automated algorithm is merely 280 (205–324) s, 6 to 12 times faster than 30–60 min reported by previous articles using Mimics or other manual software,^11^ which makes it more feasible in routine clinical practice.

Compared to intraoperative observation, the preoperative identification accuracy by surgeons using a combination of 3‐D reconstruction and chest CT scans reaches 0.85, within a median time consumption of 2 min.^1^, ^2^, ^3^ Error analysis showed that most errors occurred due to difficulties in distinguishing the merging of vessels on the proximal end. With the model improved, the accuracy may be further elevated. Due to the limitation of retrospective study, the real preoperative identification accuracy of these cases was not documented and can only be inferred by example. Massive bleeding occurred in one case due to the misdetection of a mediastinum A^4+5^ during dissection of the upper pulmonary vein (patient 16, Figure S16). Such variant accounts for about 18% of all cases, which indicates a high misdetection rate in the traditional surgery planning process. The AI algorithm has shown a generalization ability to recognize some rare variants, such as mediastinum A^4+5^ (patient 16, Figure S16), independent B^2a^ and B^2b^ (patient 7, Figure S7). However, it also showed instability in reconstruction of interlobular variants, such as V^2^ derived from the lower pulmonary vein, which was successful in patient 17 (Figure S17) but failed in patient 5 (Figure S5). Although some misclassification and misdetection were observed in the AI reconstruction, a thoracic surgeon can make an autonomous decision preoperatively (also referring to CT images) and intraoperatively rather than solely depend on the reconstruction, and they are the final arbiters. This novel fully automated approach could serve as a valuable tool to rapidly detect the evident risky variation in preoperative planning of lung segmentectomy. However, it is still necessary for thoracic surgeons to improve their interpretation ability of CT images.

To our knowledge, this is the first study which has attempted to validate the performance of an automated 3‐D reconstruction algorithm based on noncontrast CT scans. The algorithm showed equivalent accuracy with the traditional method and may synchronize with 2‐D imaging to allow faster and more accurate identification of anatomical structures preoperatively. Admittedly, there are still some limitations regarding the current AI model. The model may not accurately determine the origin of small distal vessels, including the hybridization of arteries and veins in some complicated cases. On the one hand, we are still helping expand the training set to further improve the overall performance of the AI model. On the other hand, three‐dimensional reconstruction is an intuitive tool, which means that reliance on misleading reconstruction results could induce misjudgment on anatomical structure. Therefore, we will continue improving this model, as well as cautiously apply it to our clinical practice.

In conclusion, automated 3D reconstruction algorithm in itself achieves similar accuracy in both vessel and bronchial detection and classification compared to the manual approach. As a complement to CT scans, the addition of AI reconstruction renders high identification accuracy in a surprisingly short time frame. Such an algorithm may assist in the surgical planning process of segmentectomy.

## CONFLICT OF INTERESTS

DW W, HJ Z, EY L and TZ report being paid employees of the Institute of Advanced Research, Infervision Medical Technology Co., Ltd. The other authors have no conflicts of interest to declare.

## Supporting information


**Figure S1** The intraoperative observation of enrolled 20 patients and the 3‐D reconstruction by AI and manual approach.

**Table S1** Supplementary Tables.
