# Supplementary material for: A fully automated noncontrast CT 3‐D reconstruction algorithm enabled accurate anatomical demonstration for lung segmentectomy
Source: Thorac Cancer. 2022 Feb 9;13(6):795–803. doi: 10.1111/1759-7714.14322 (PMC8930461; doi:10.1111/1759-7714.14322)

Fig S1-Patient 1

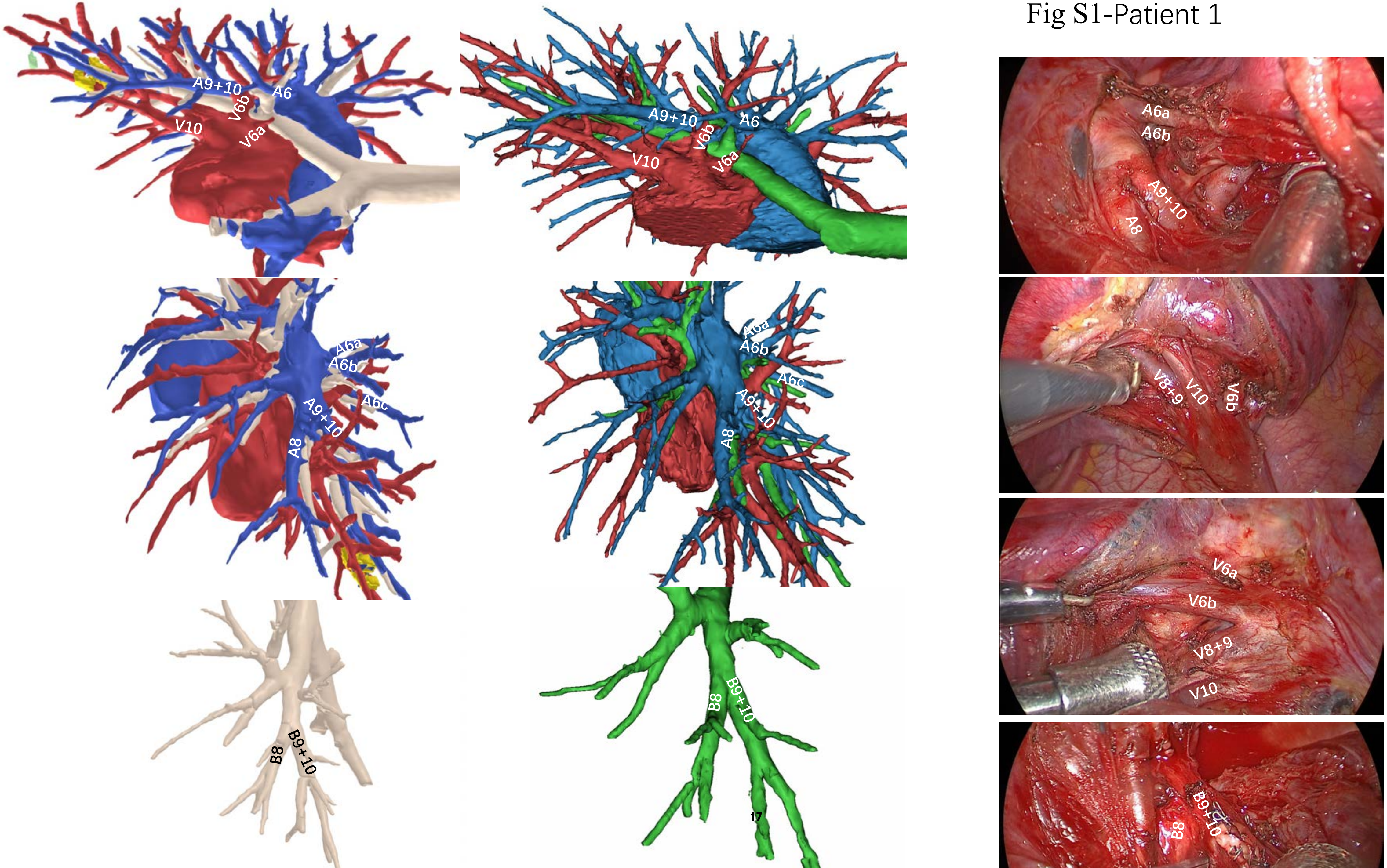

Fig S2-Patient 2

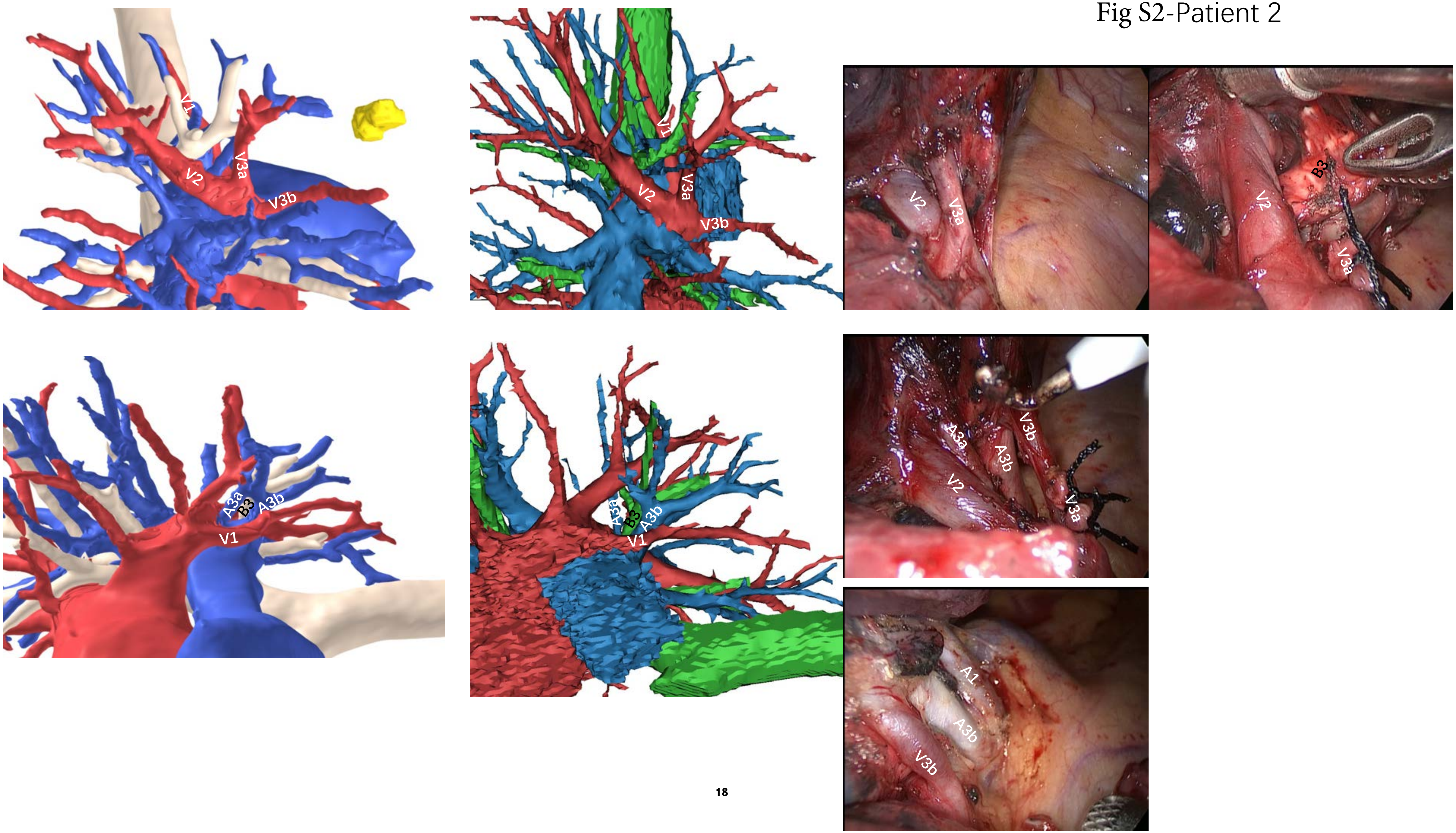

Fig S3-Patient 3

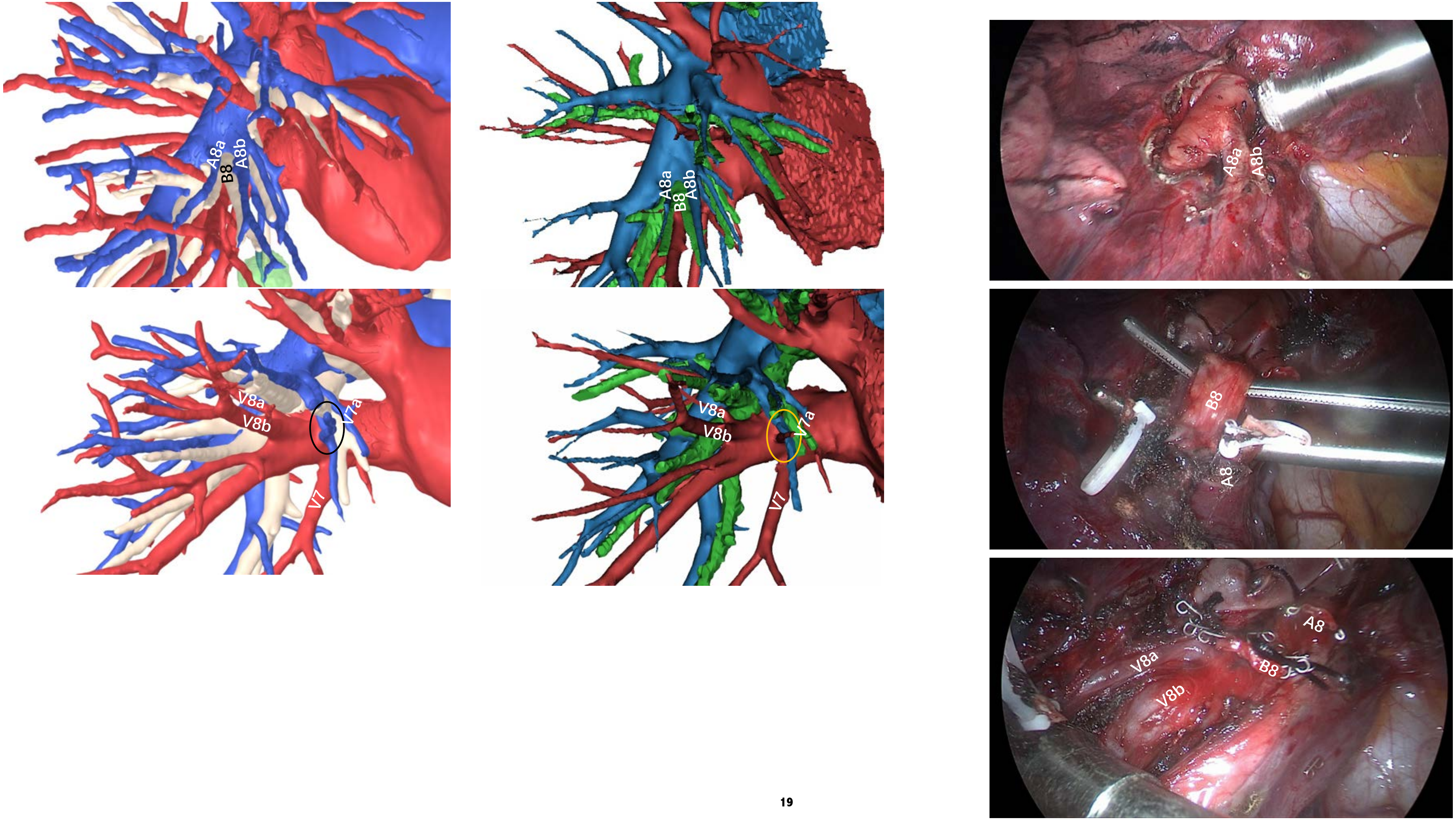

Fig S4-Patient 4

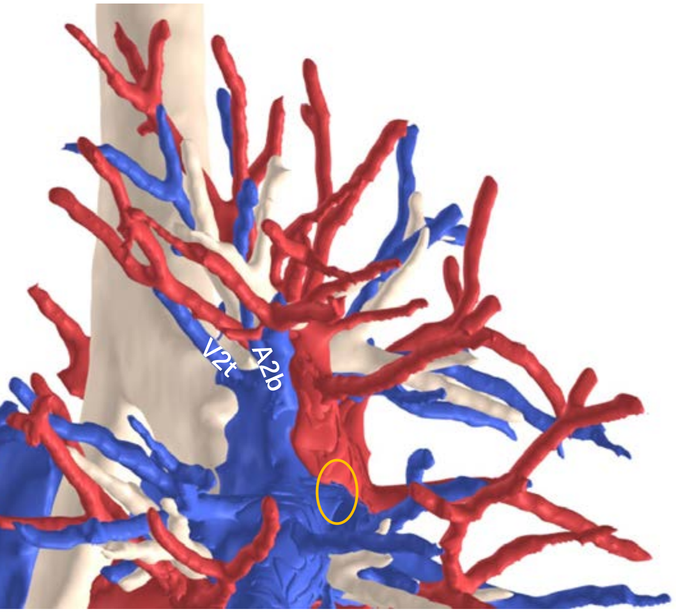

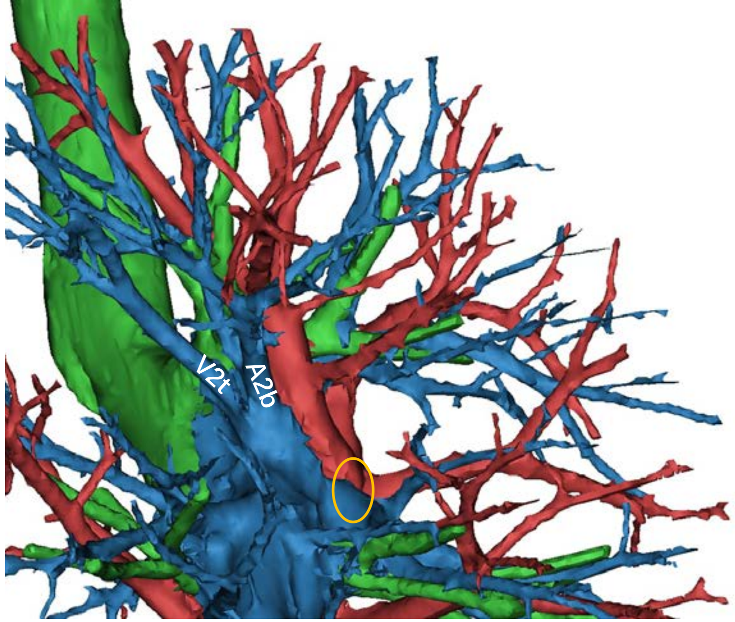

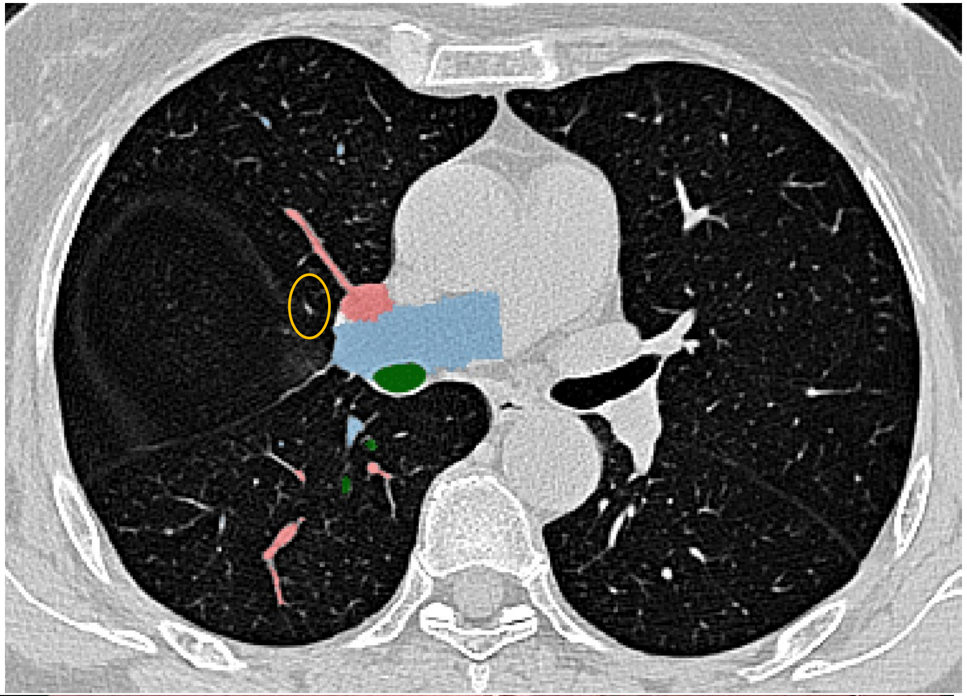

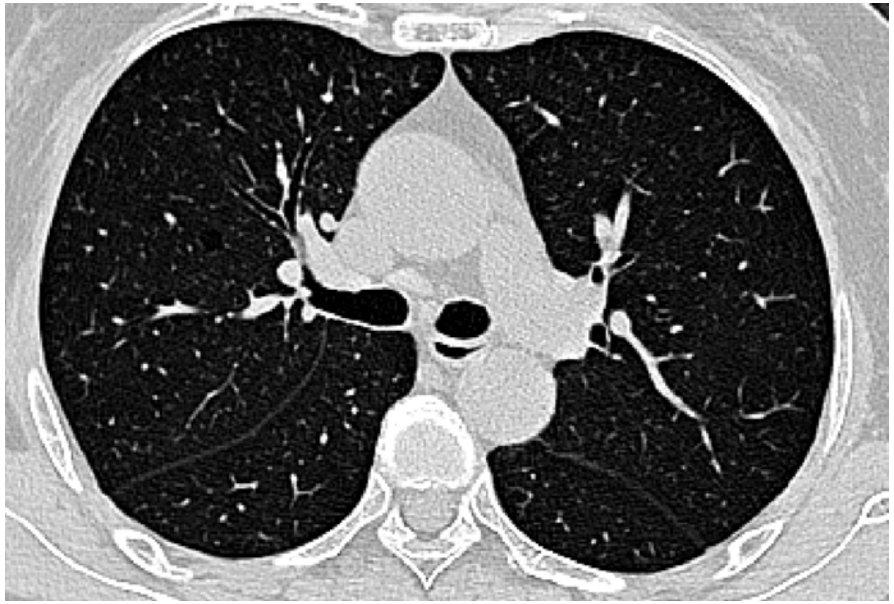

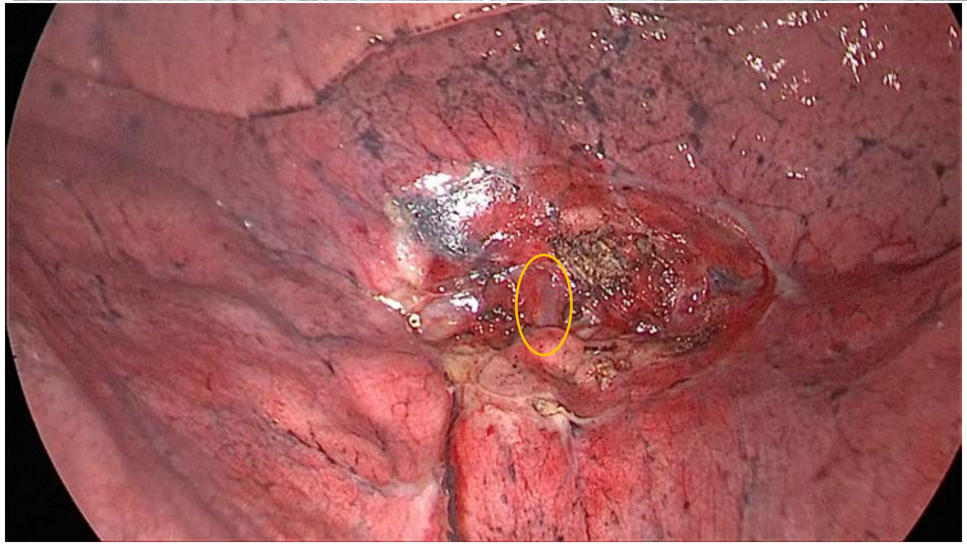

Fig S5-Patient 5

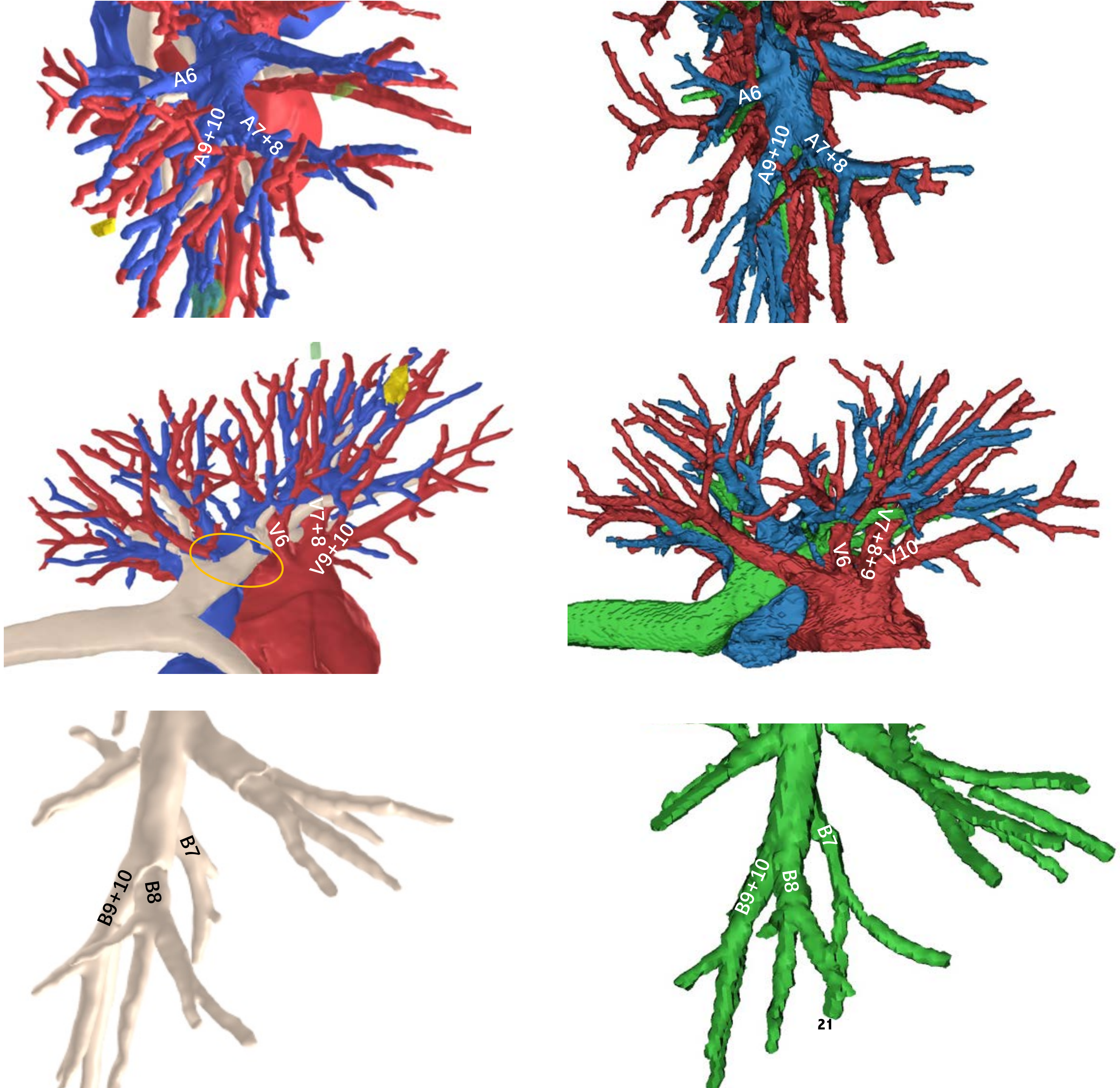

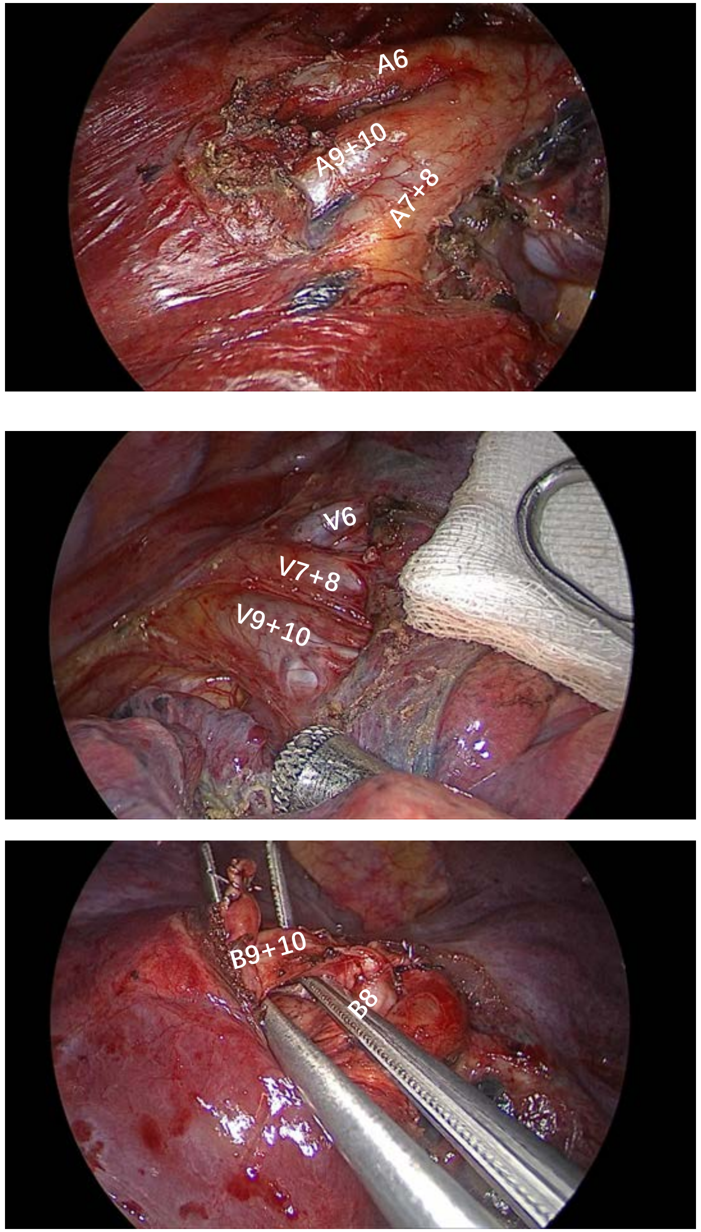

Fig S6-Patient 6

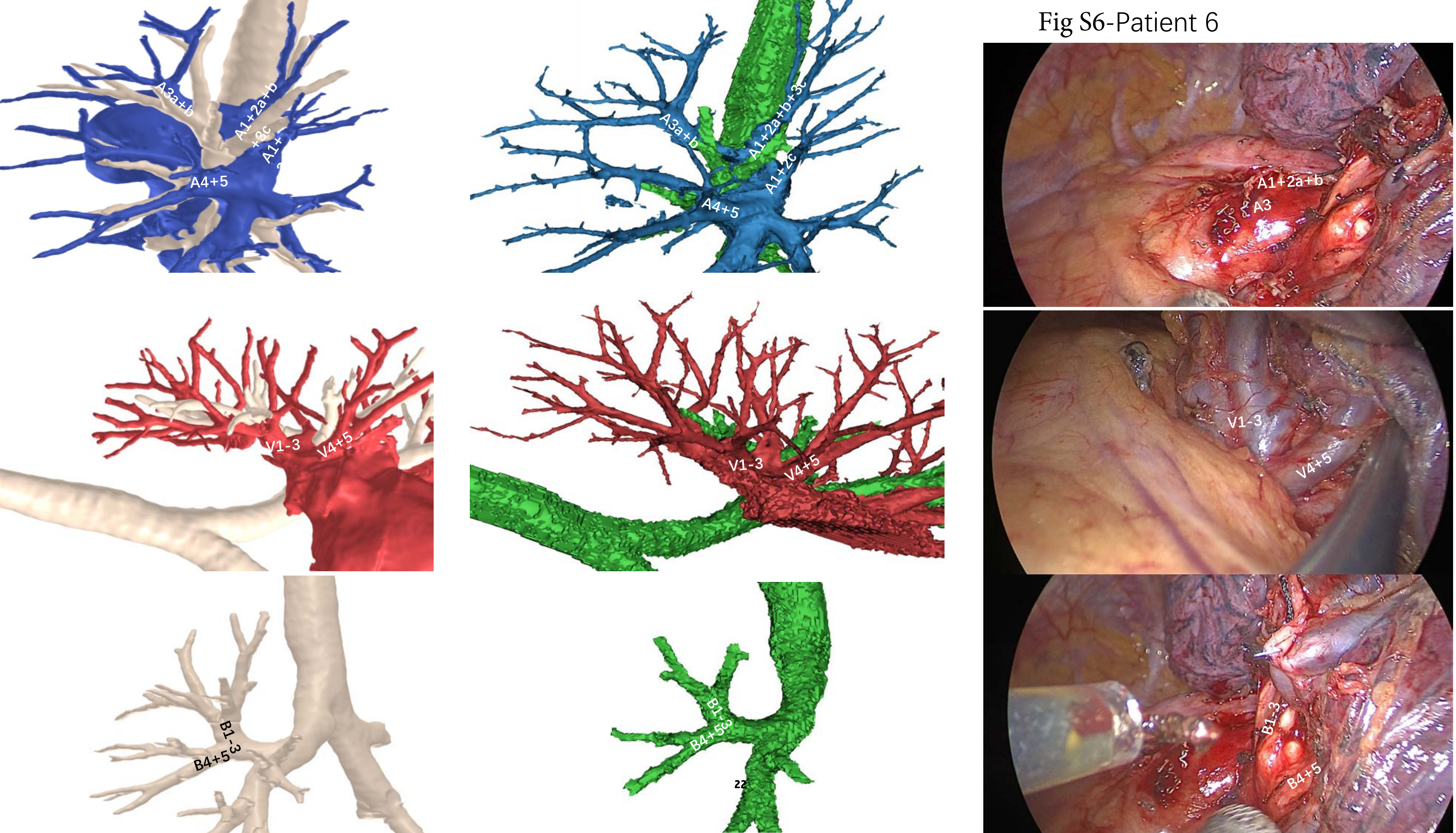

Fig S7-Patient 7

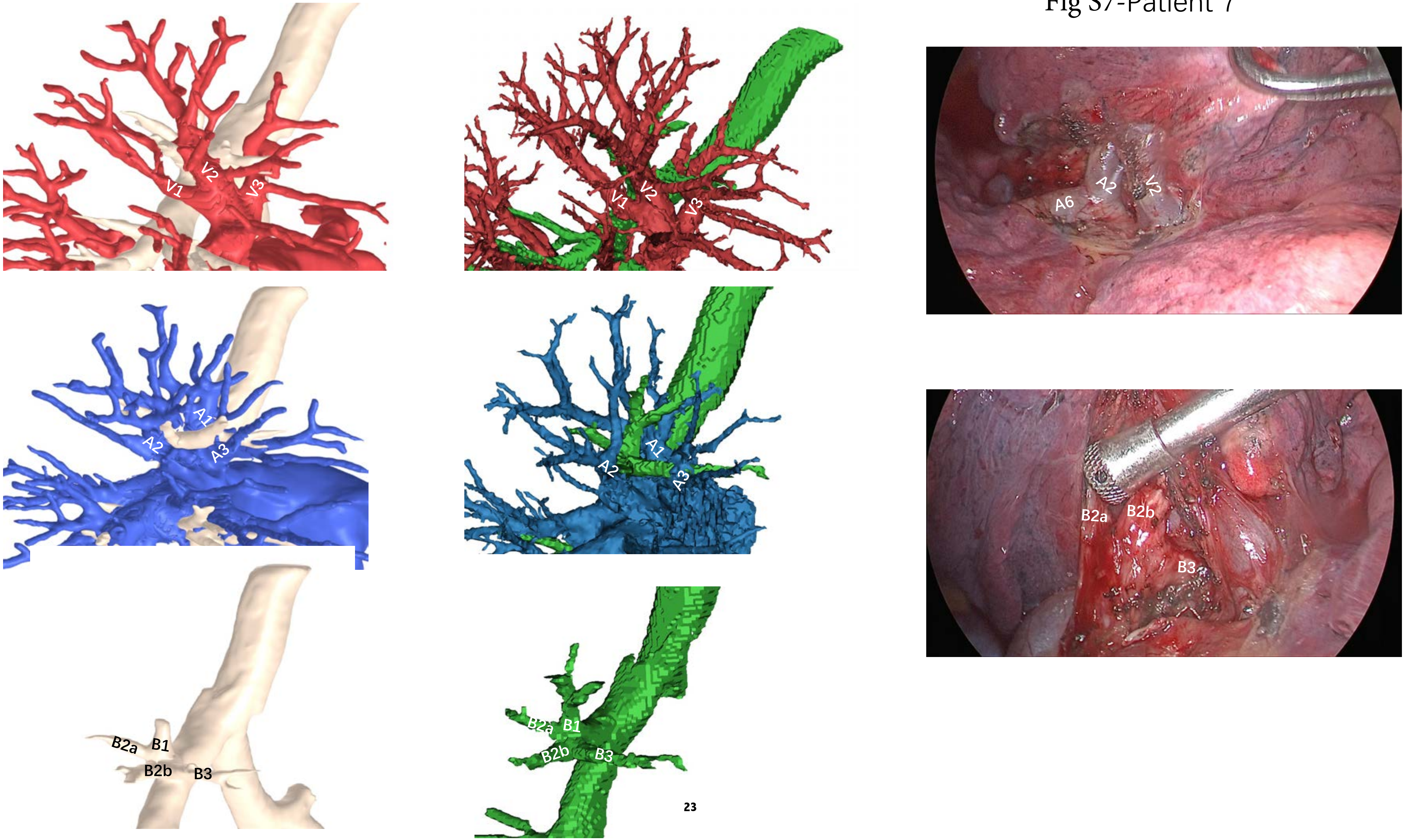

Fig S8-Patient 8

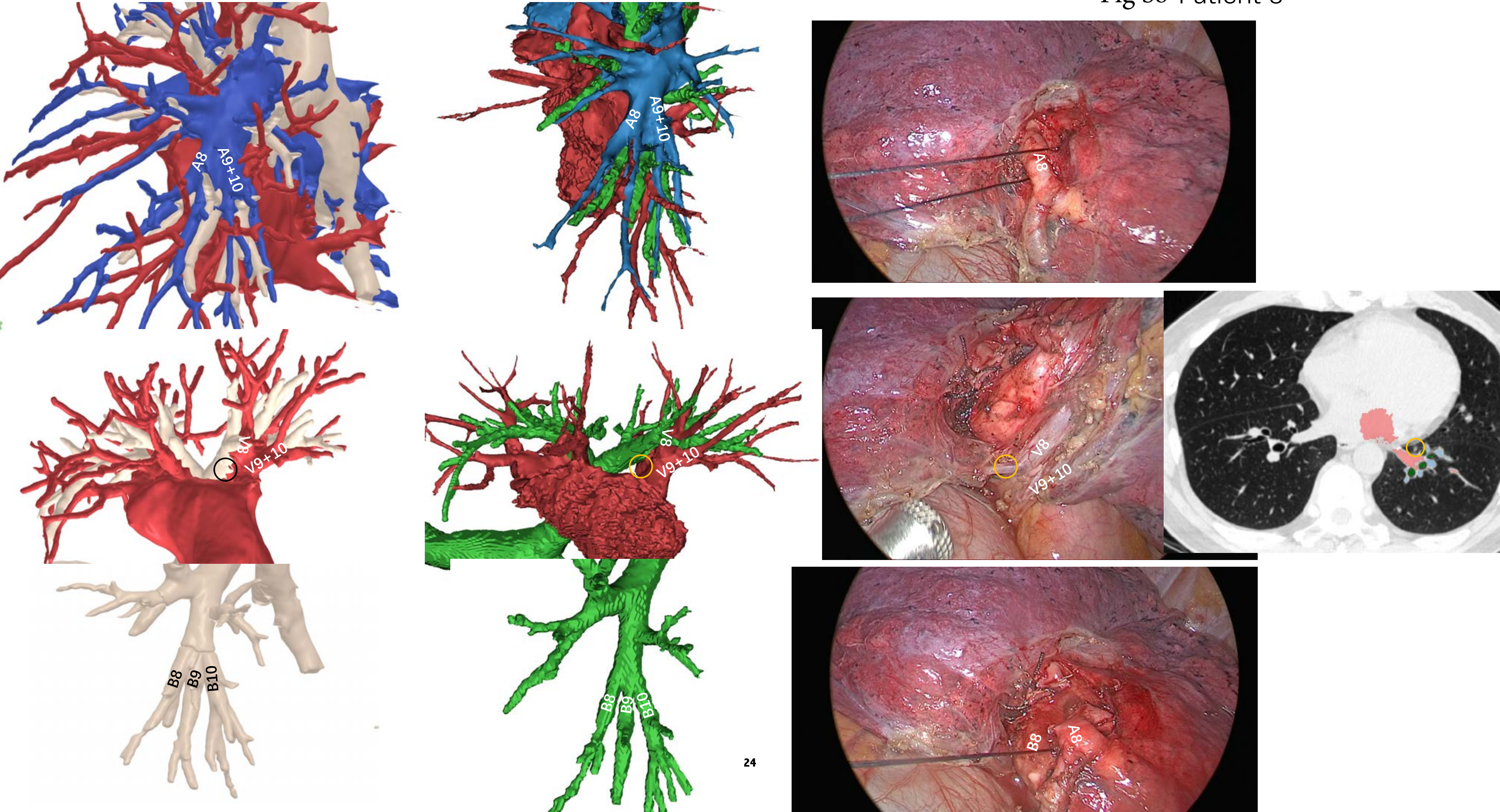

Fig S9-Patient 9

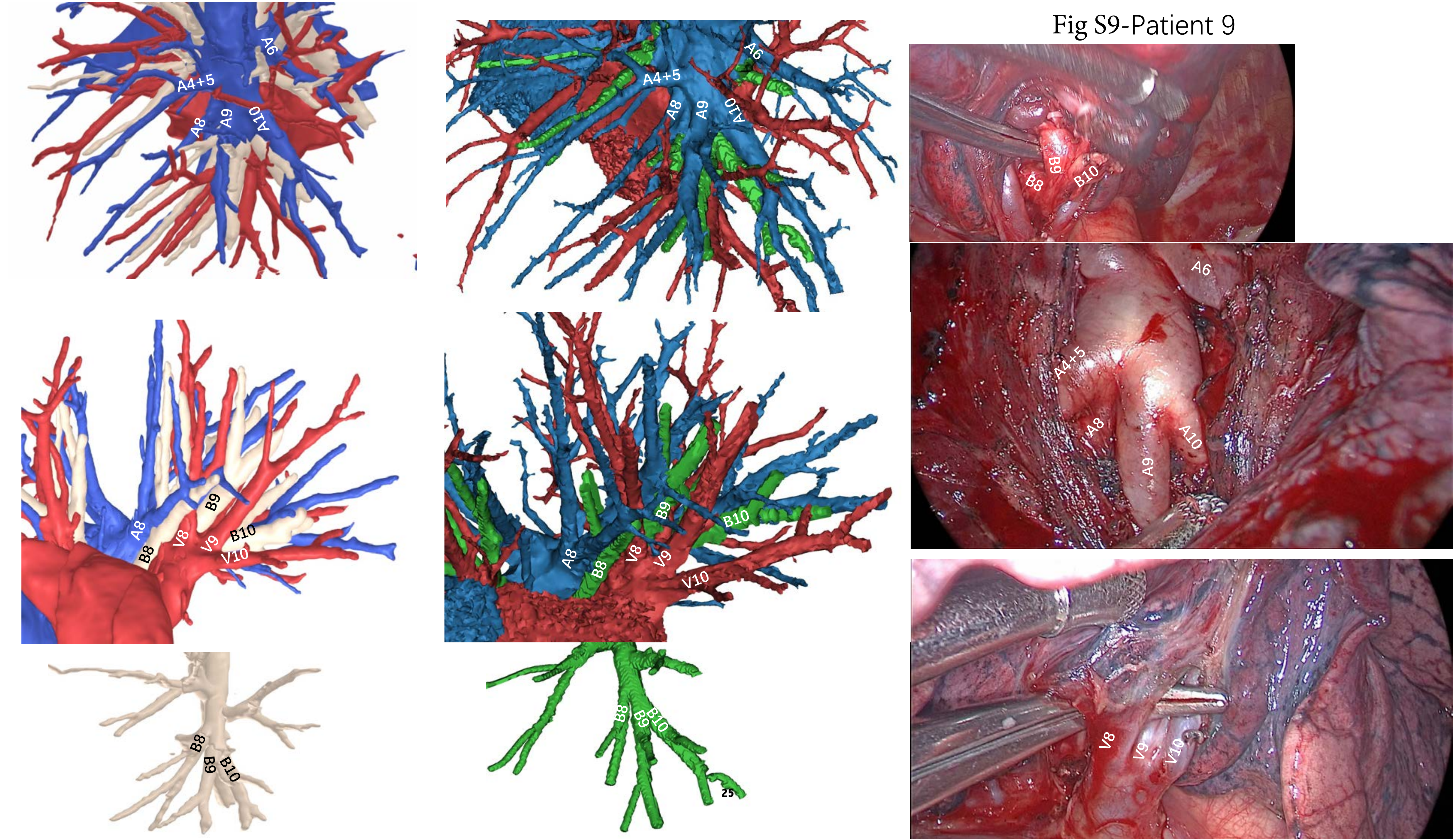

Fig S10-Patient 10

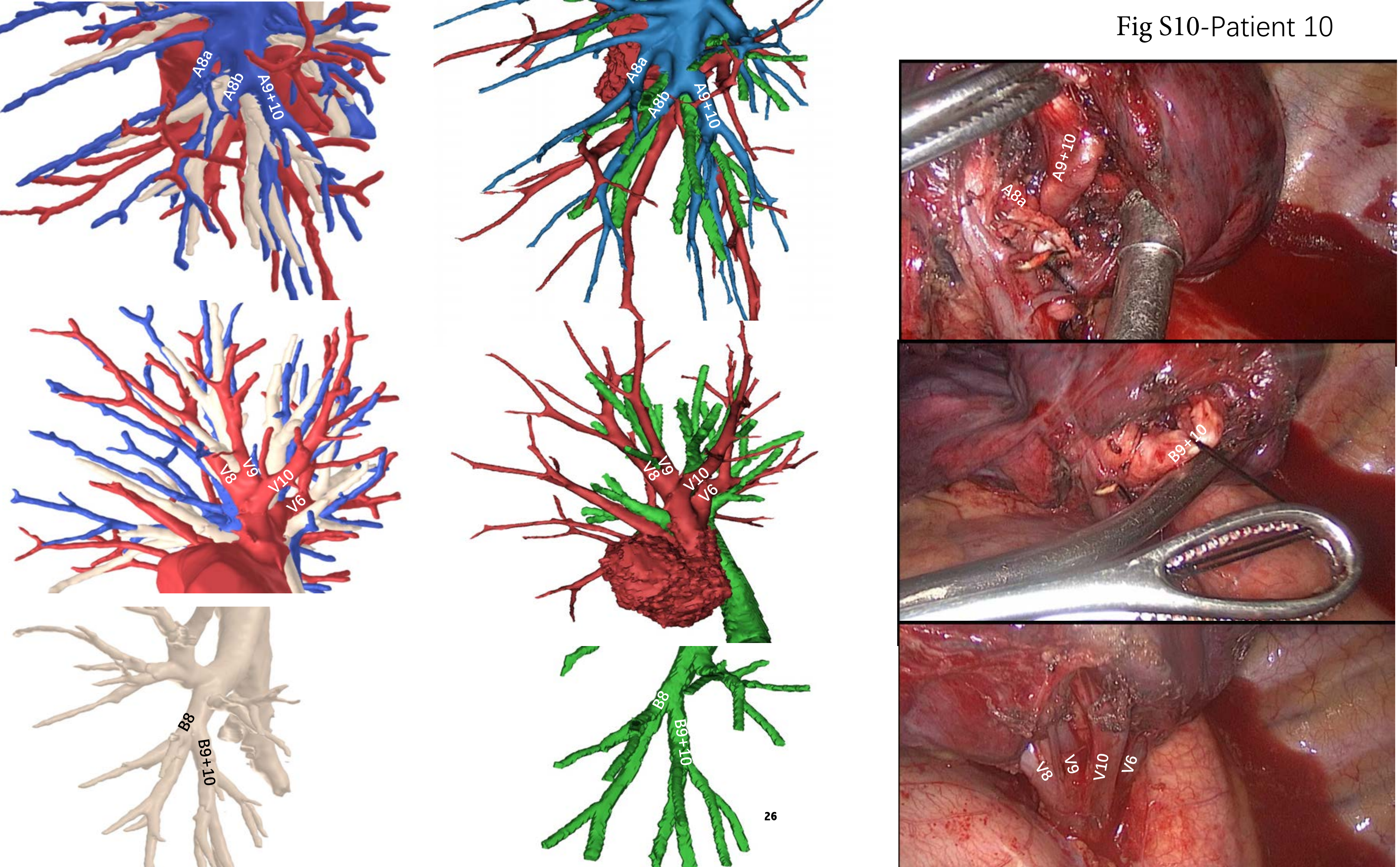

Fig S11-Patient 11

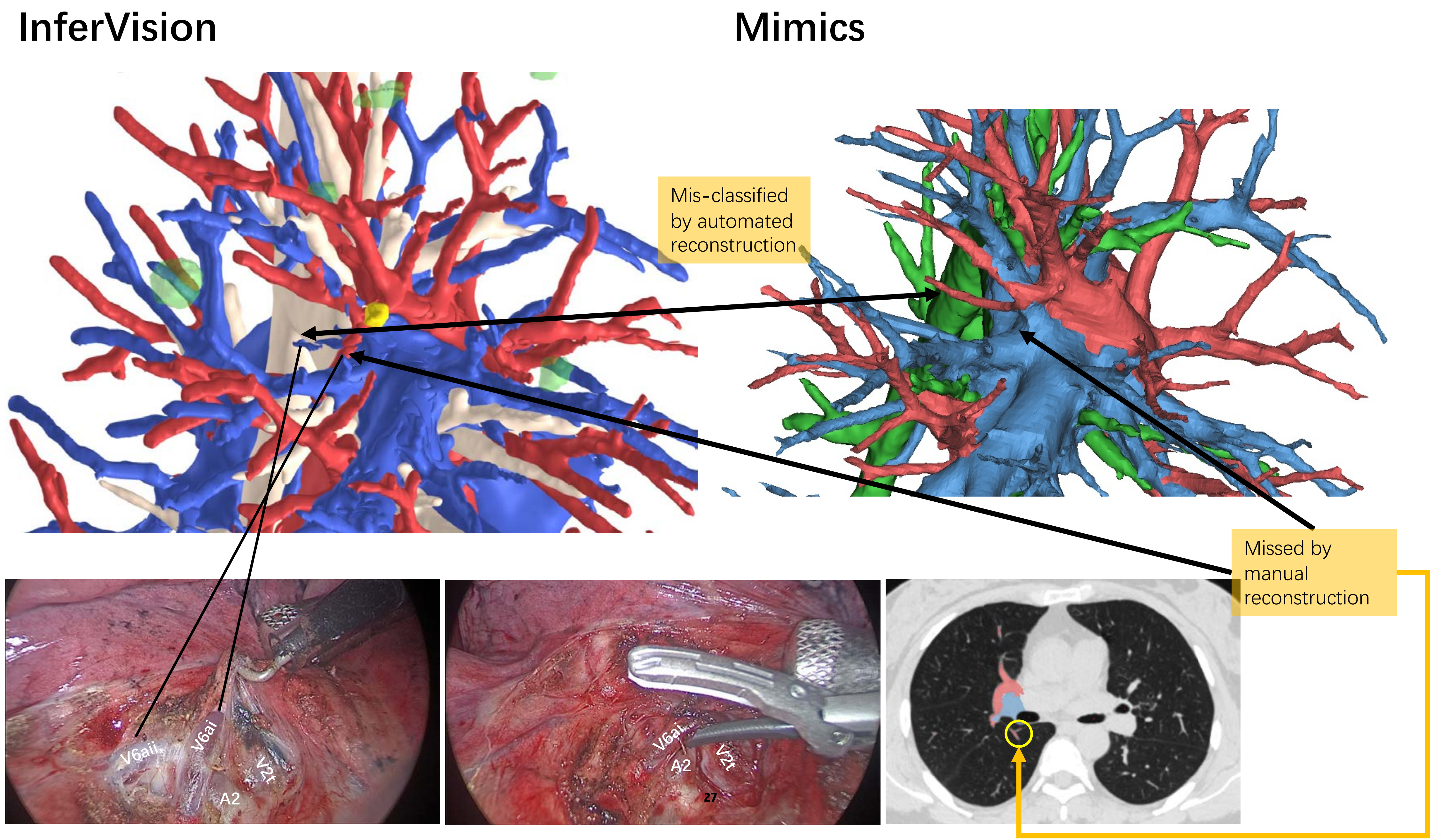

InferVision

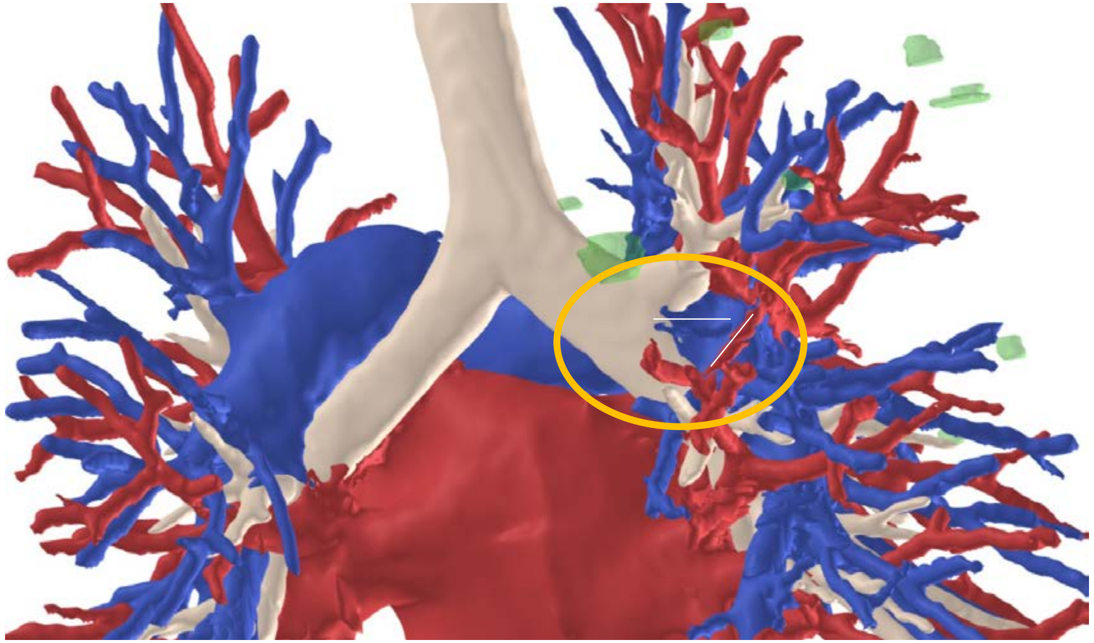

Mimics

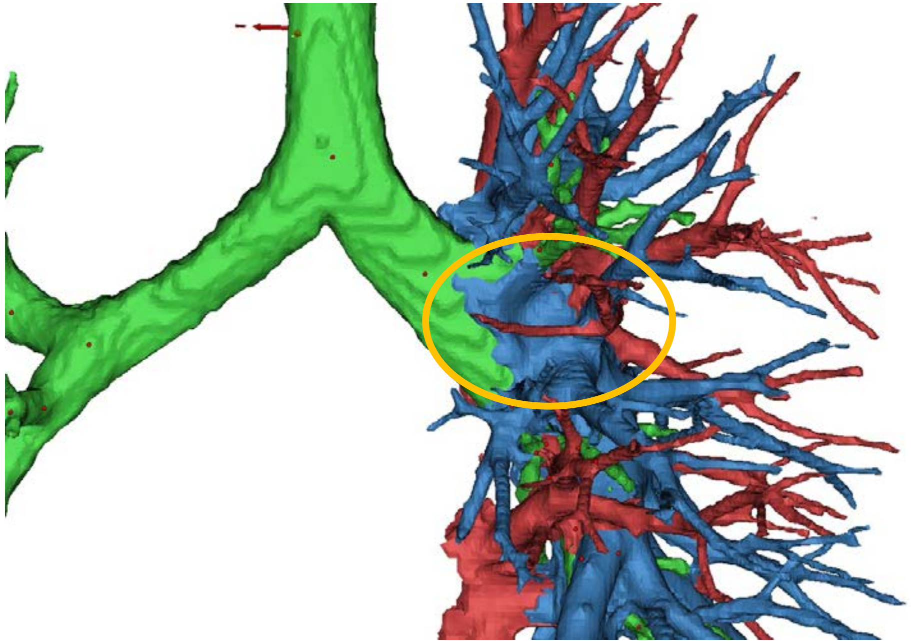

Fig S12-Patient 12

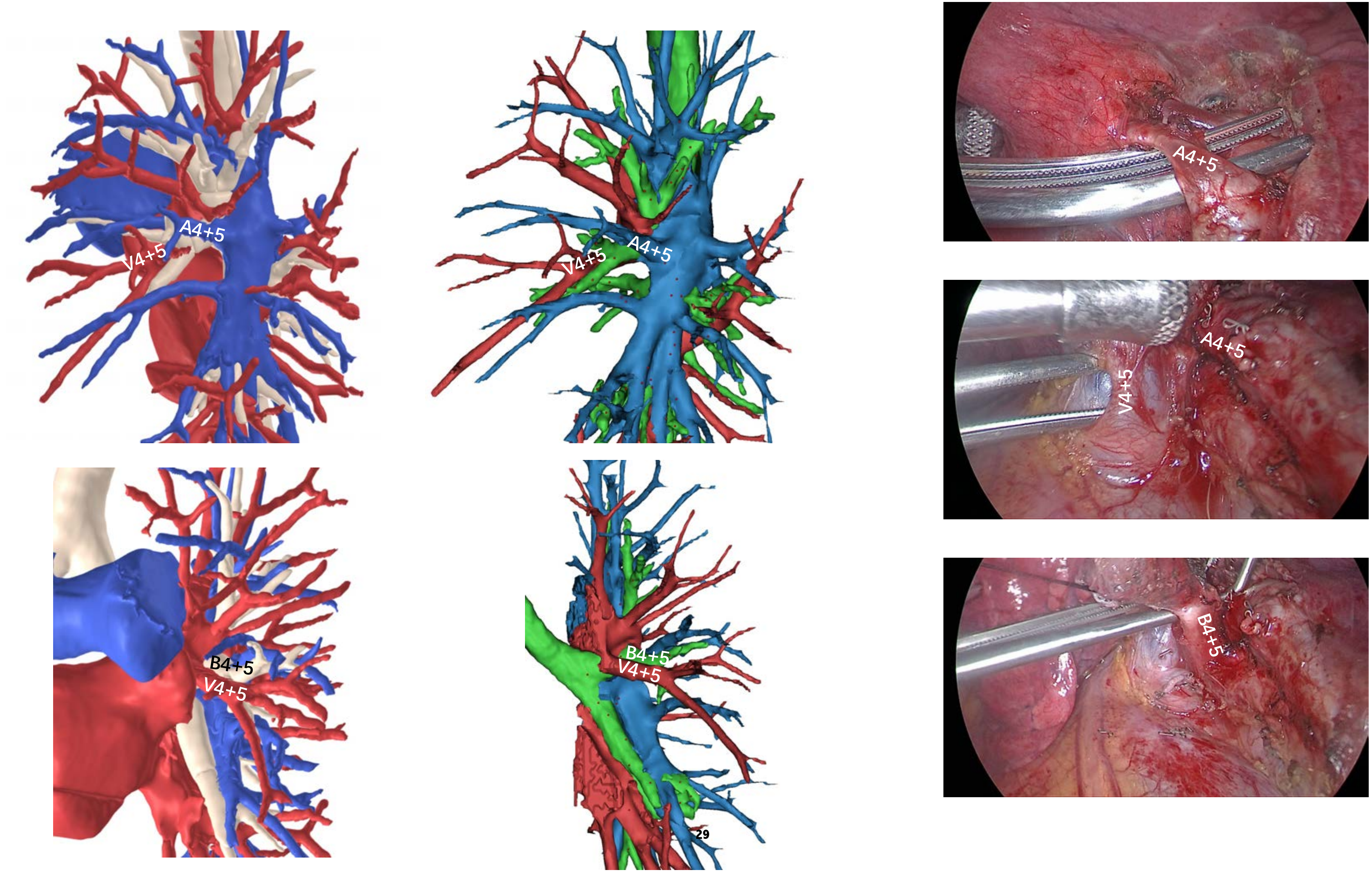

Fig S13-Patient 13

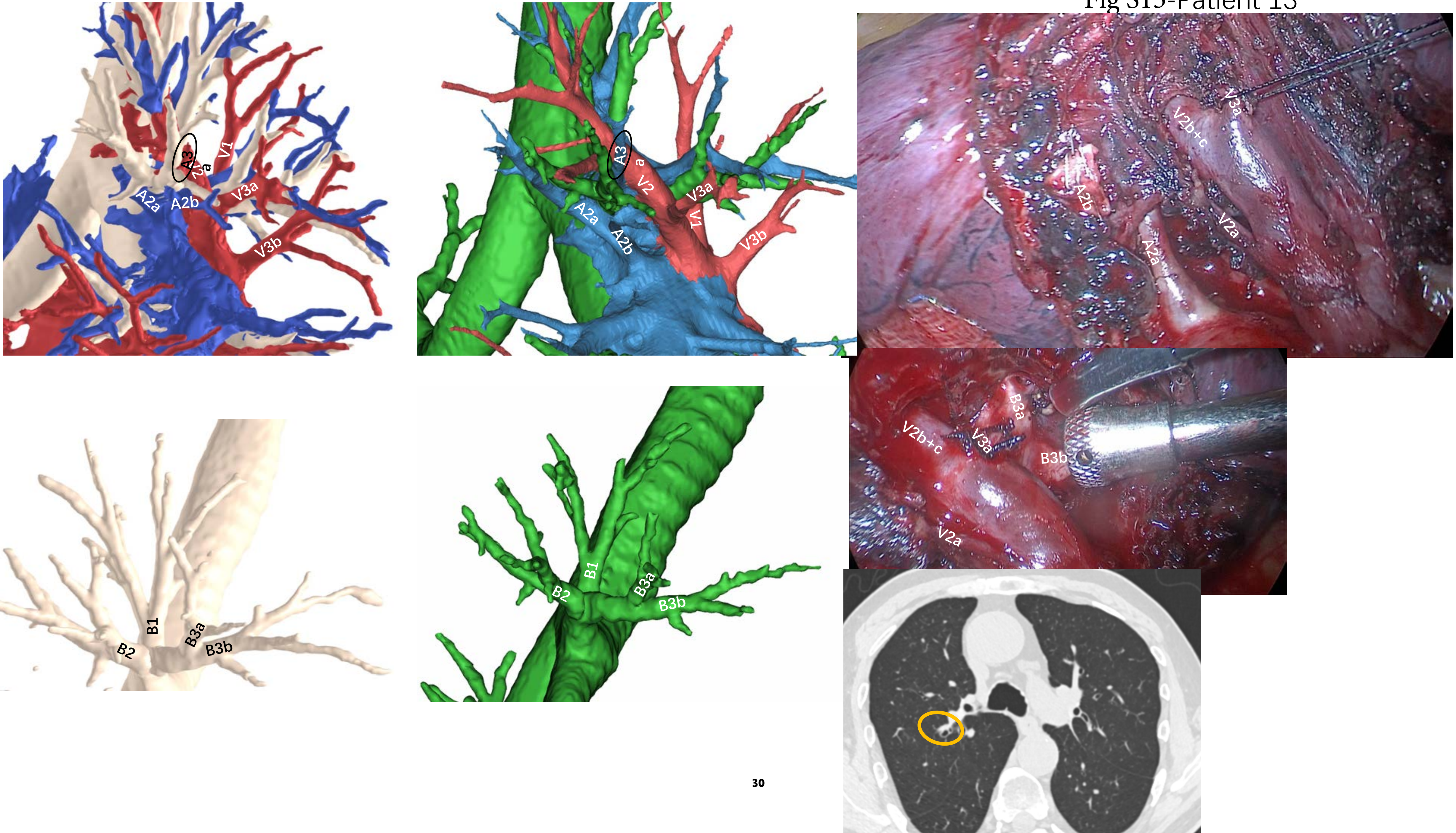

Fig S14-Patient 14

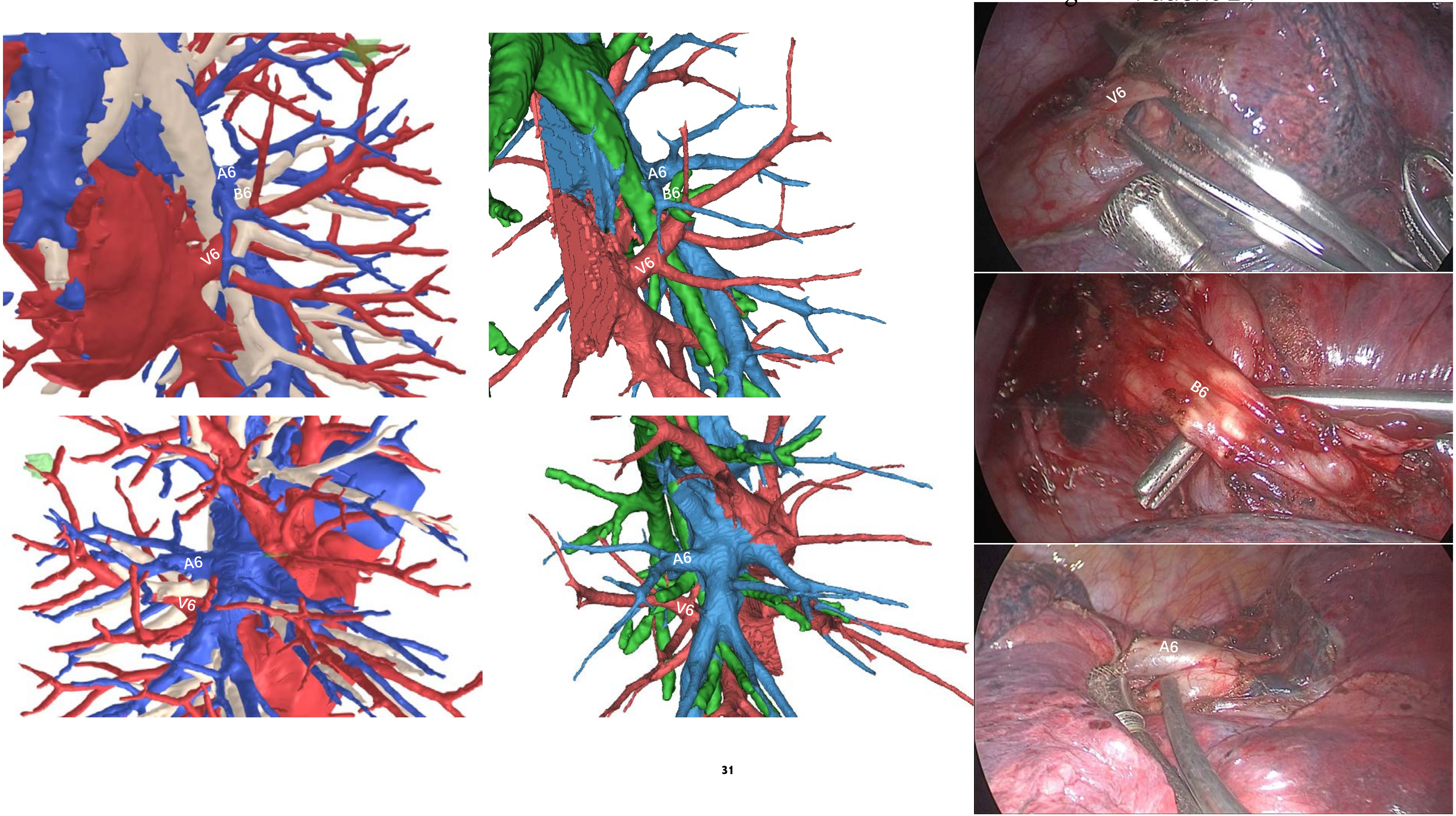

Fig S15-Patient 15

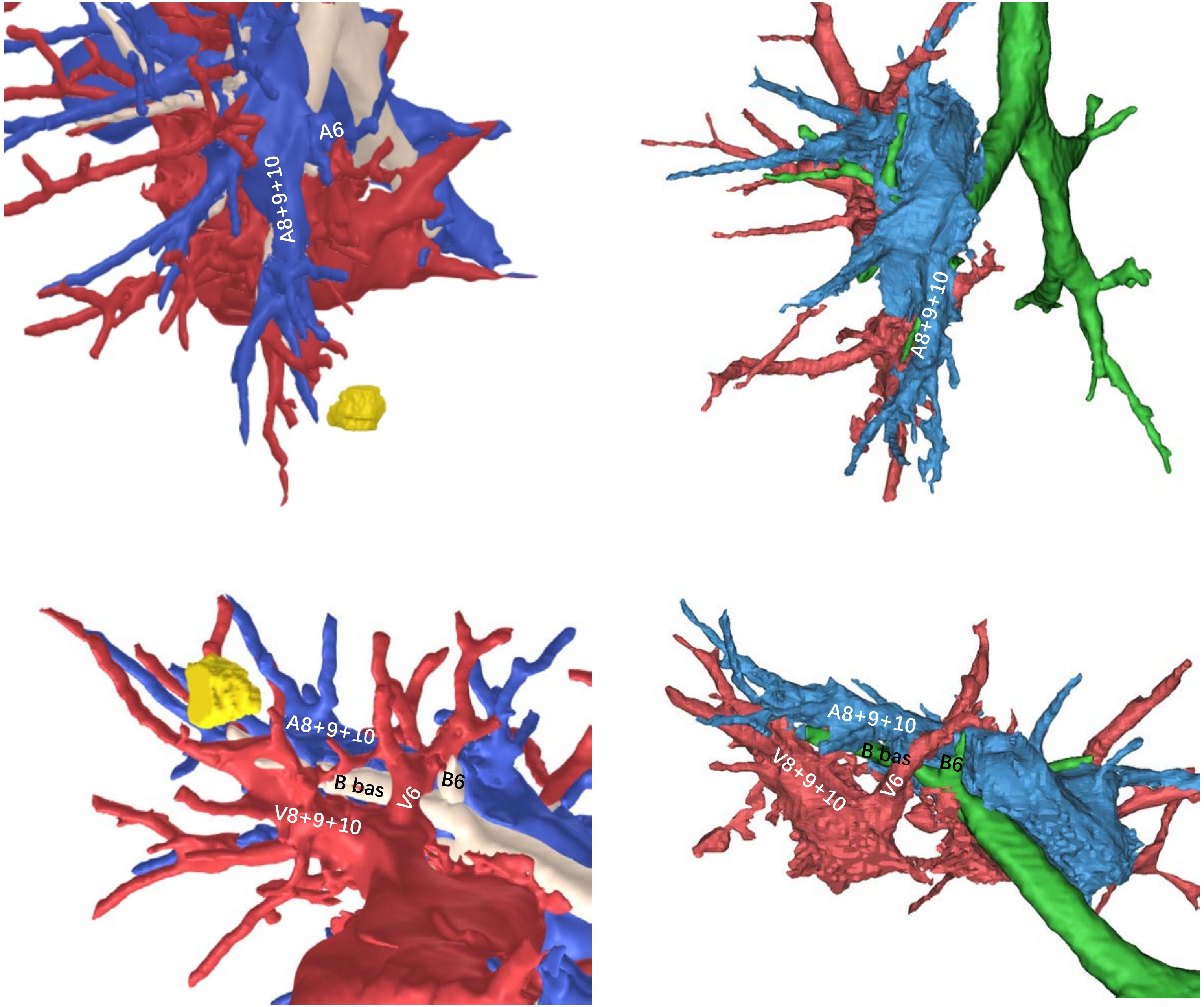

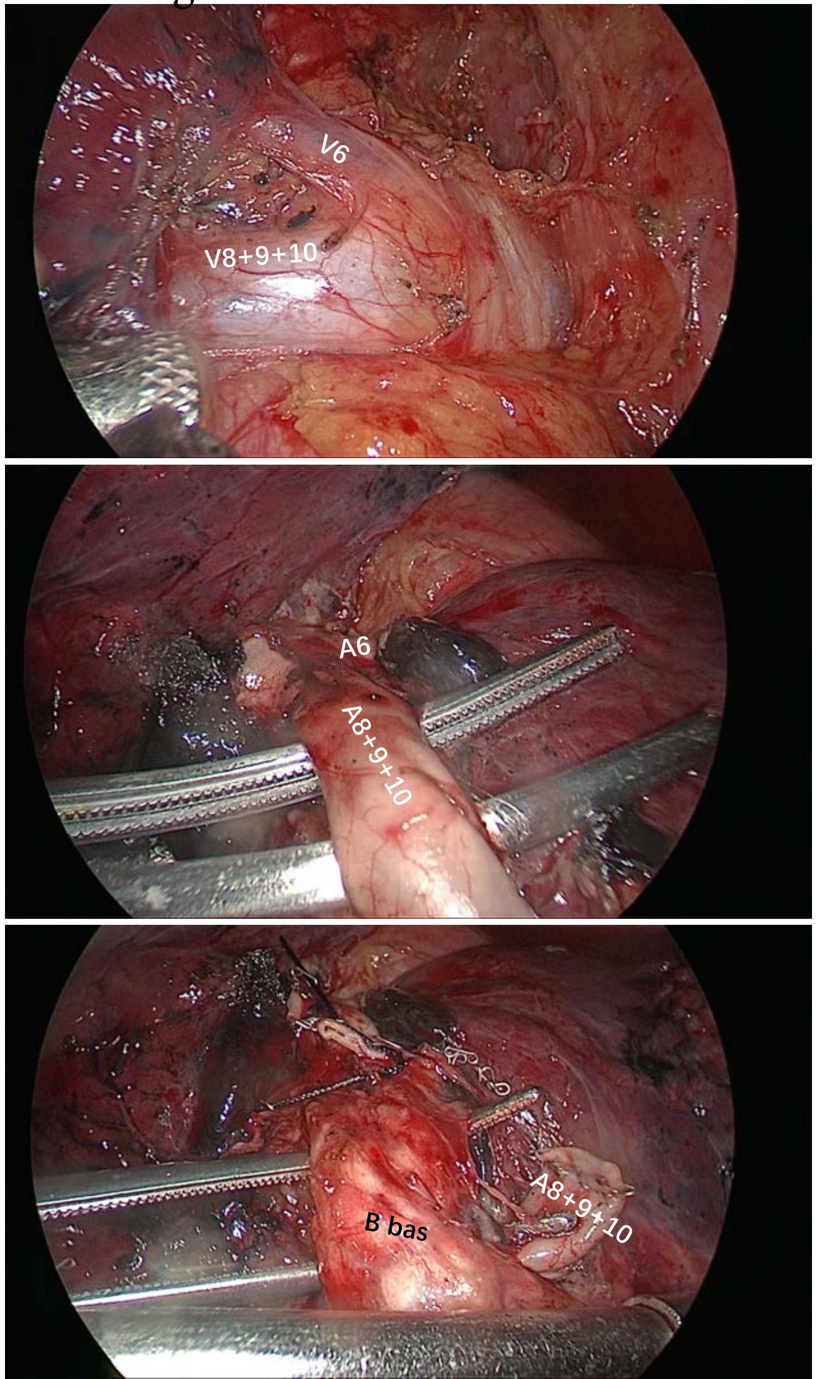

Fig S16-Patient 16

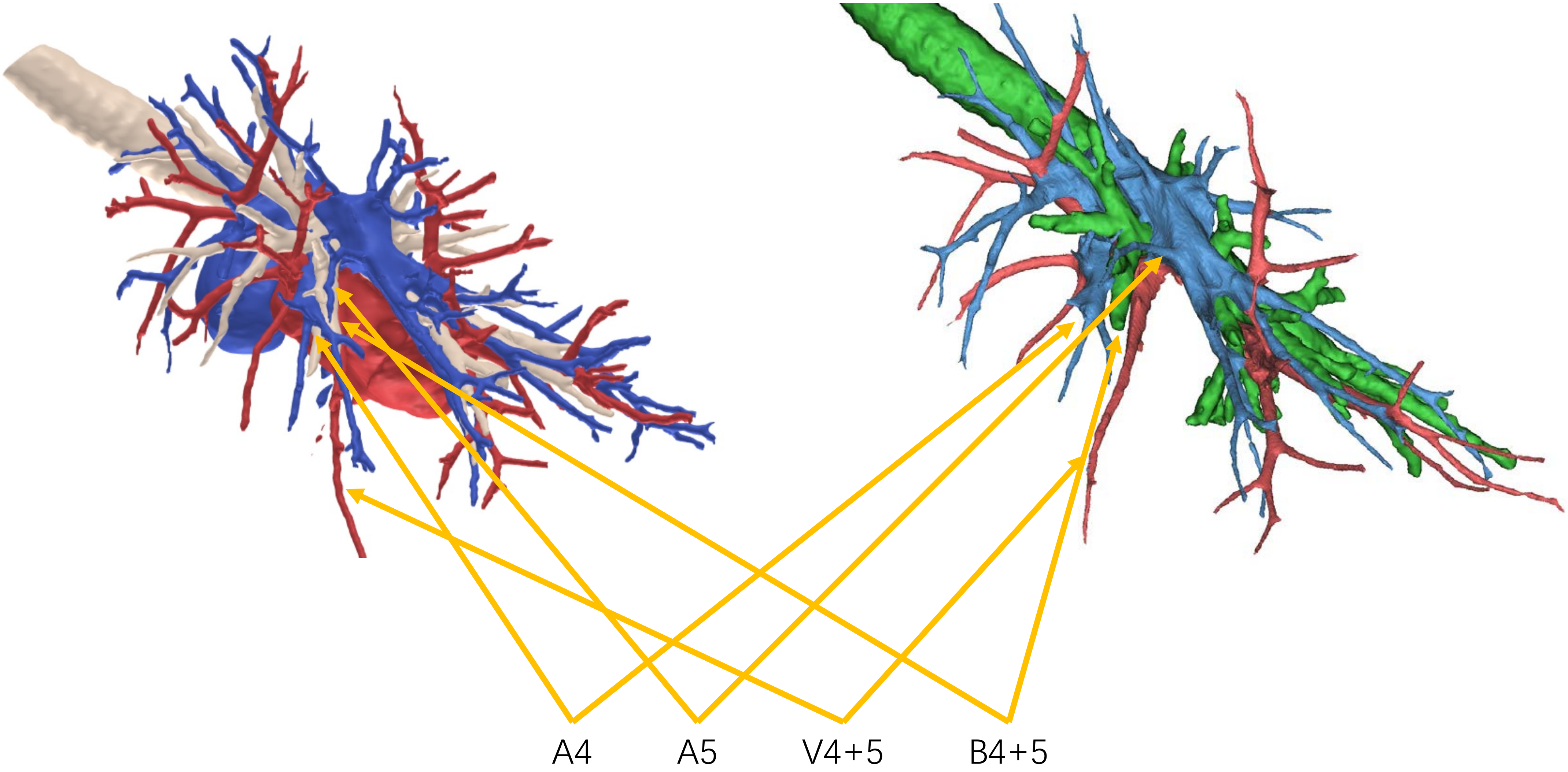

Fig S16-Patient 16

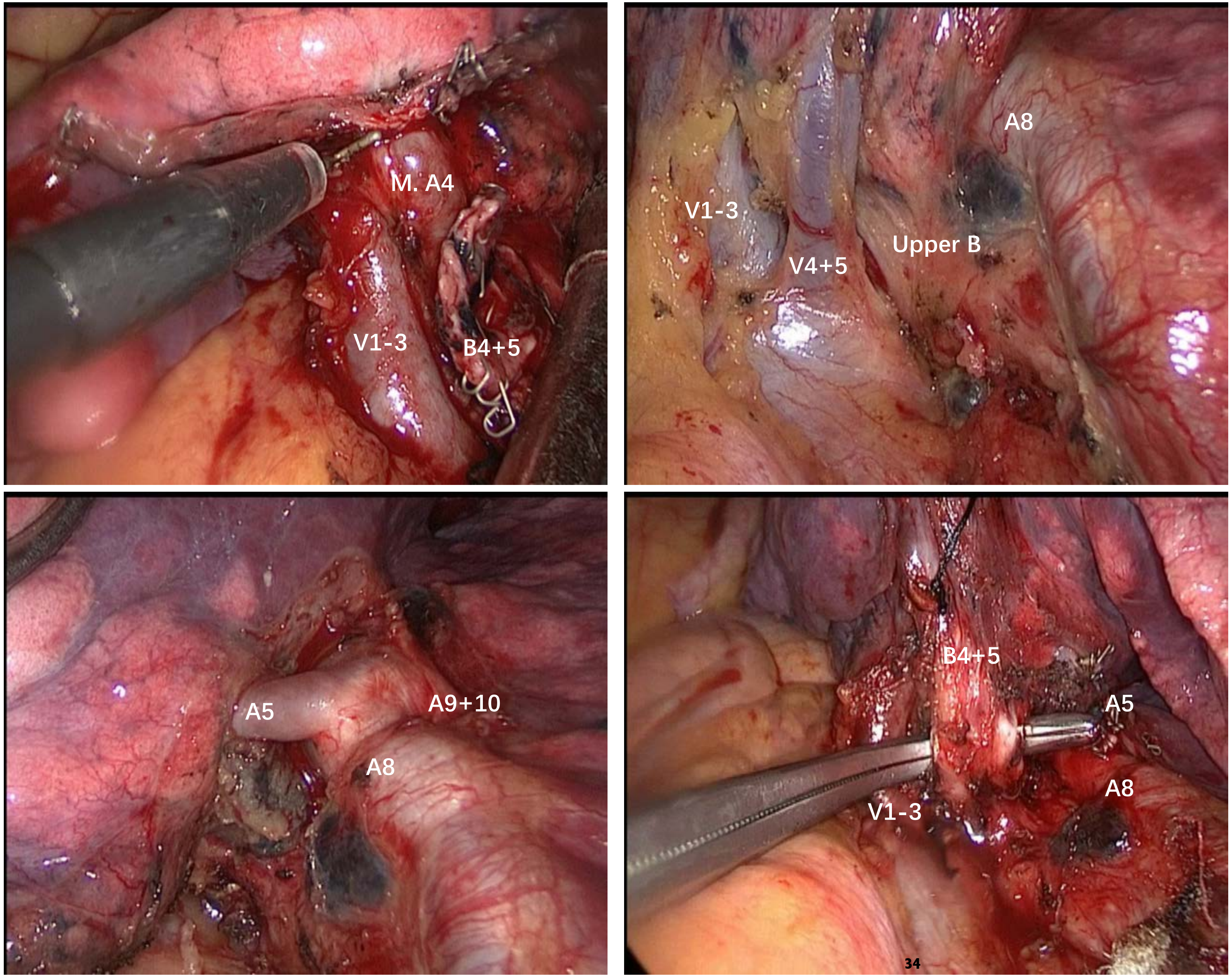

Fig S17-Patient 17

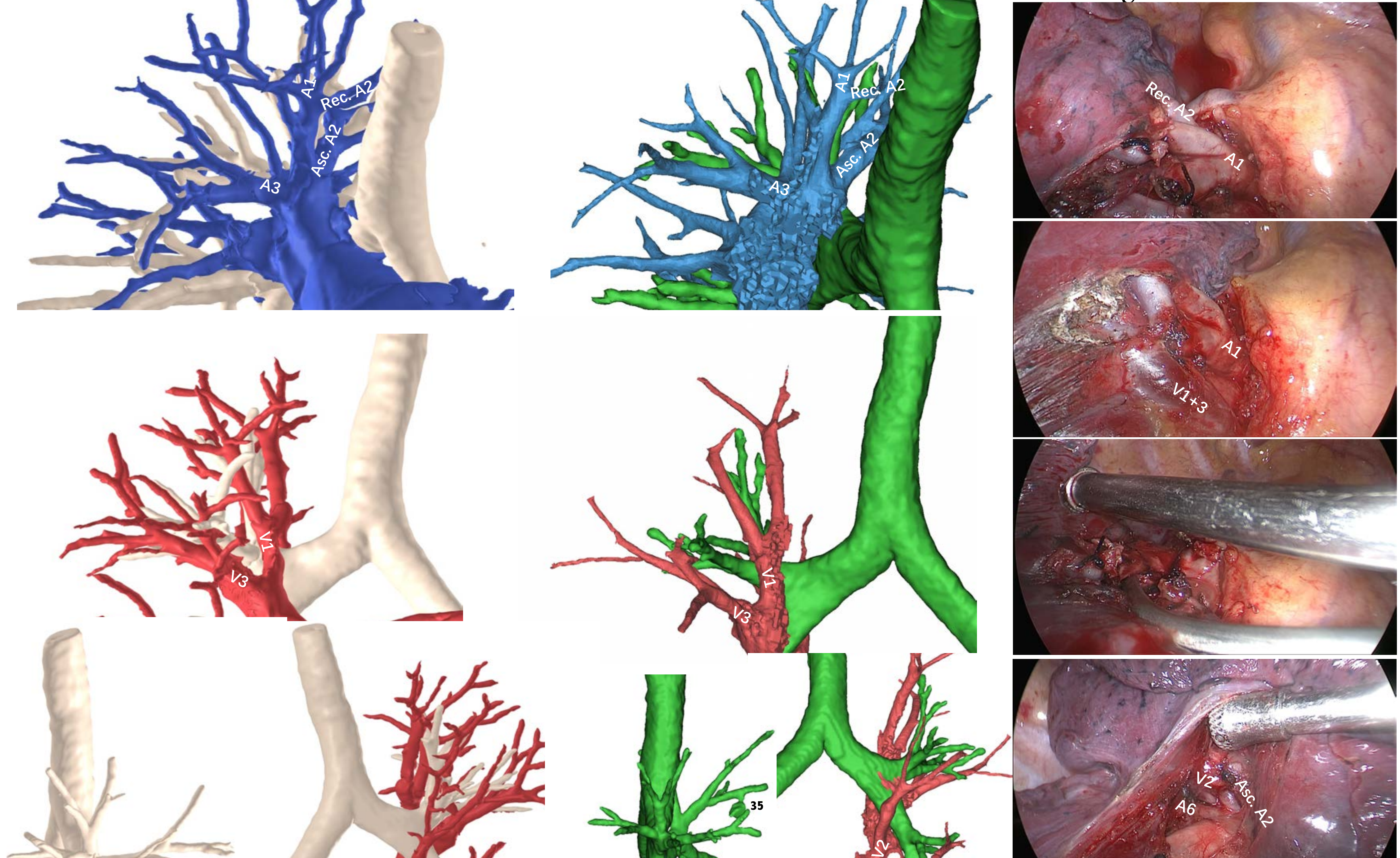

Fig S18-Patient 18

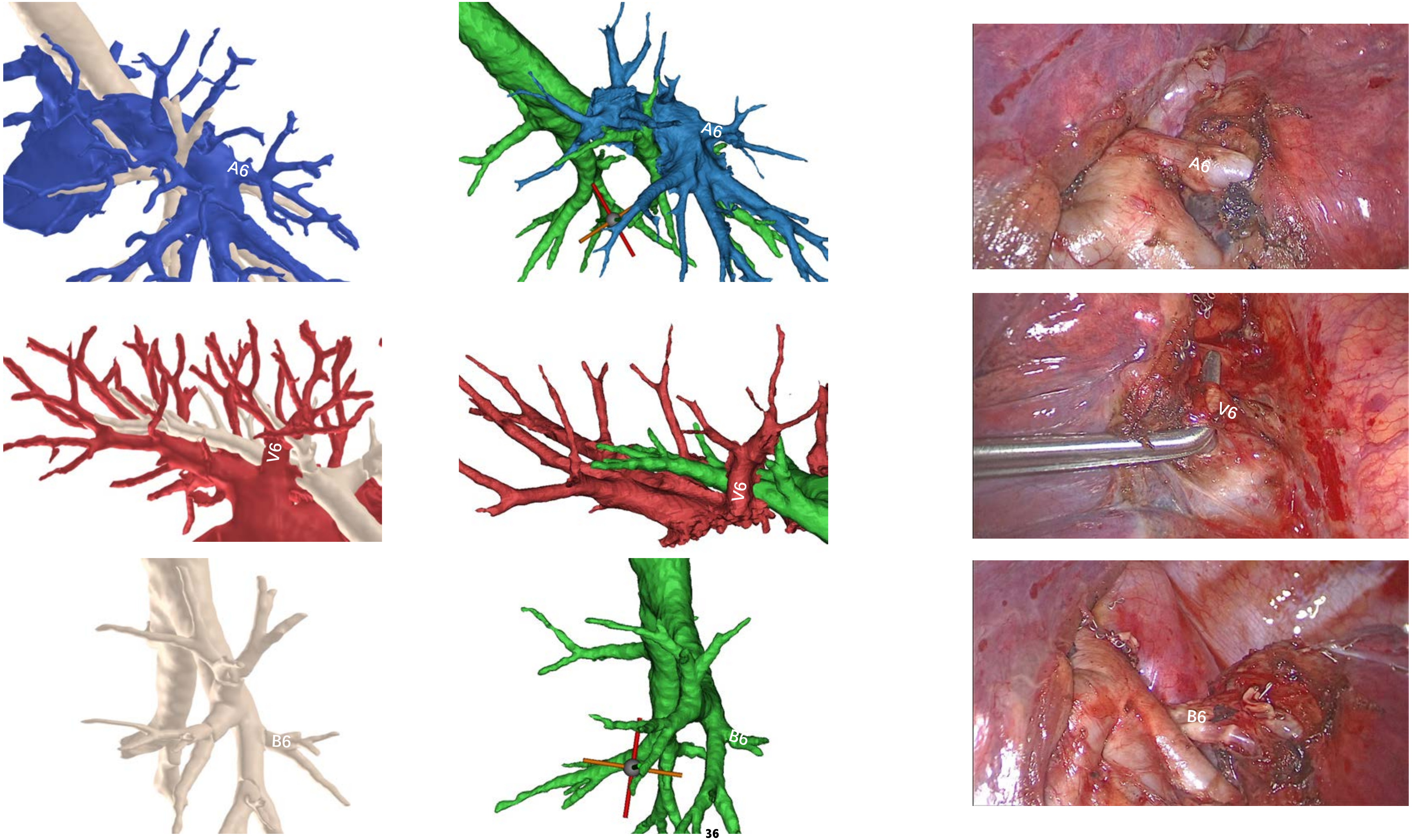

Fig S19-Patient 19

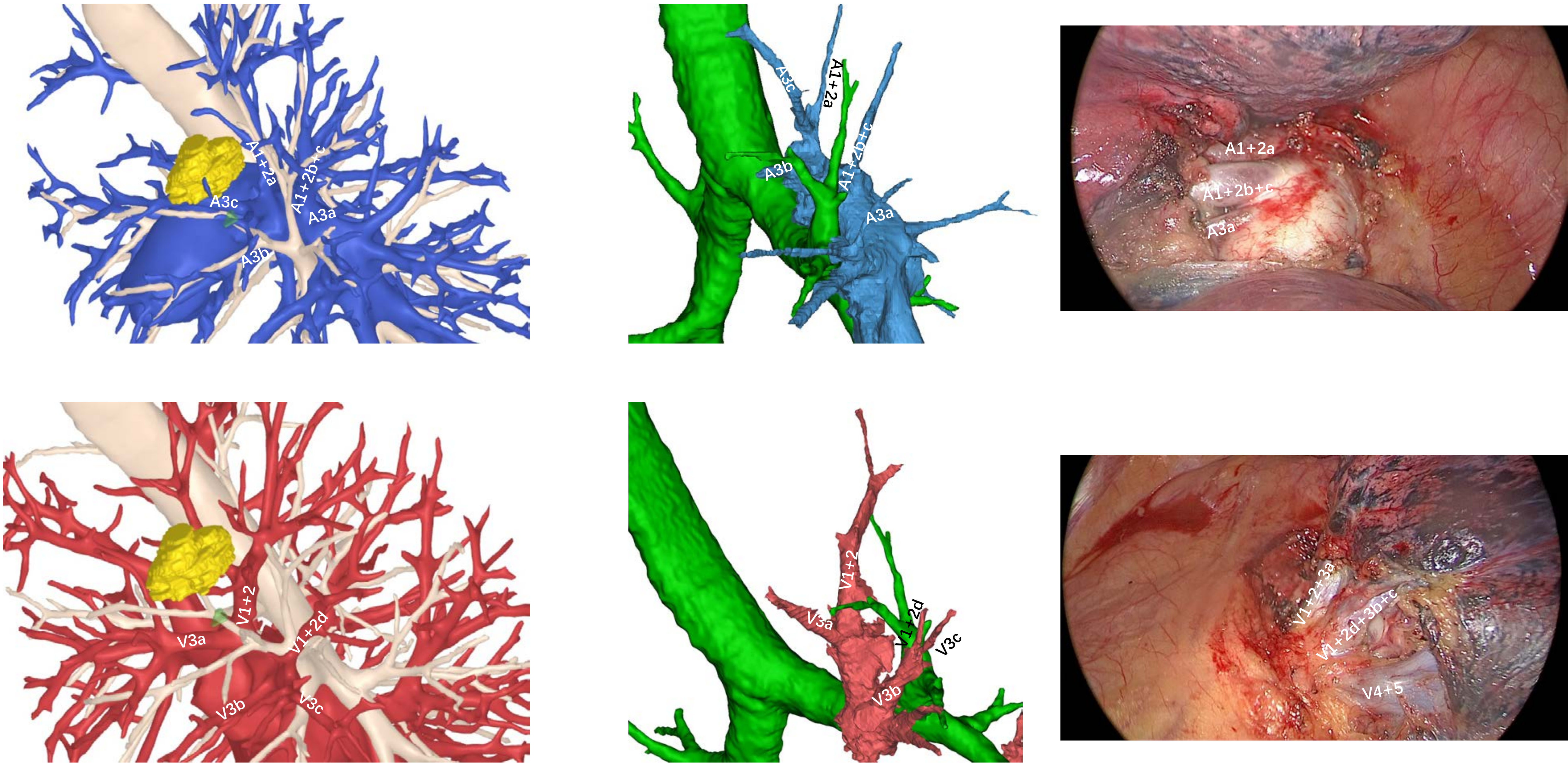

Fig S20-Patient 20

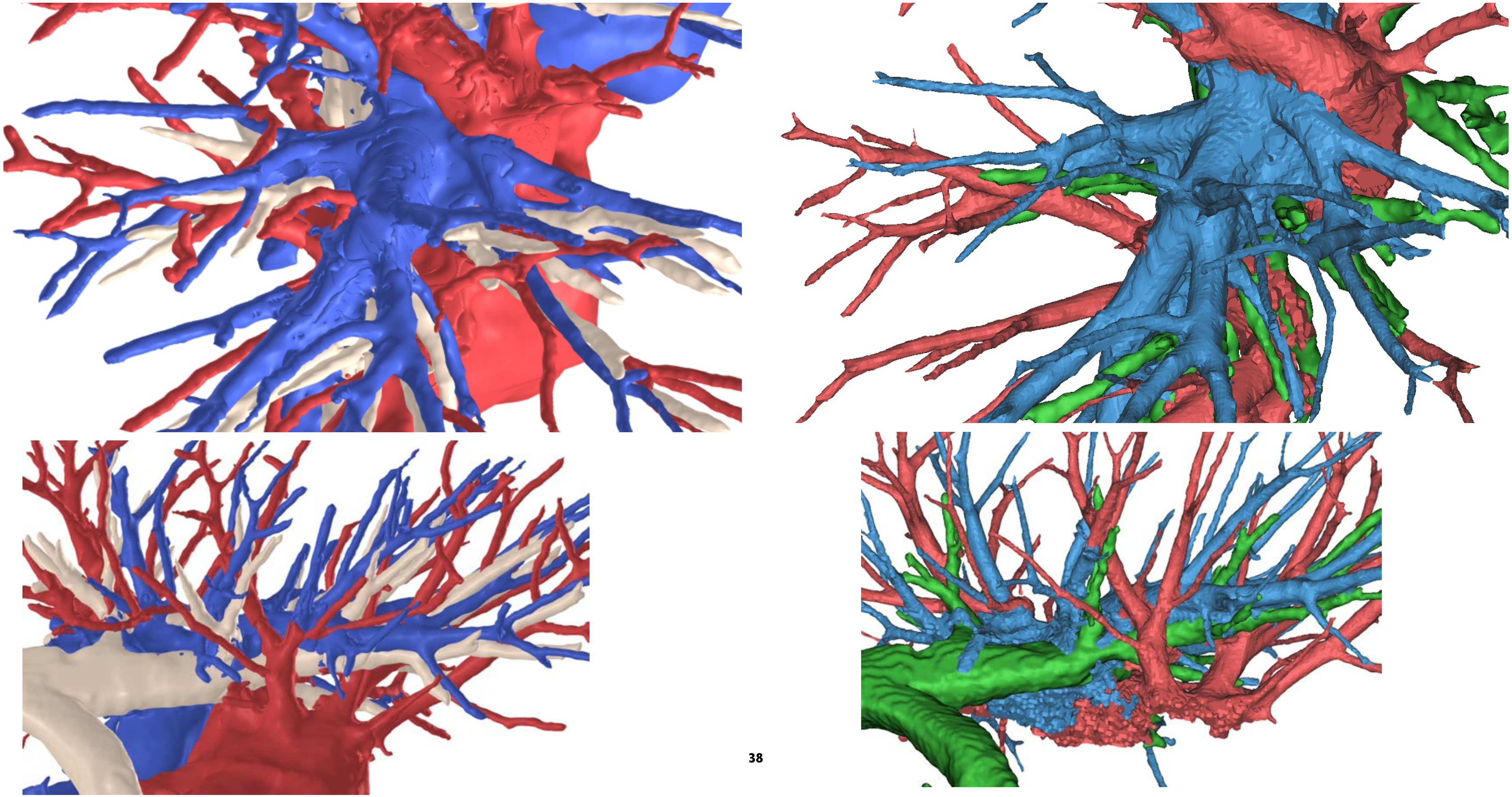

Fig S20-Patient 20

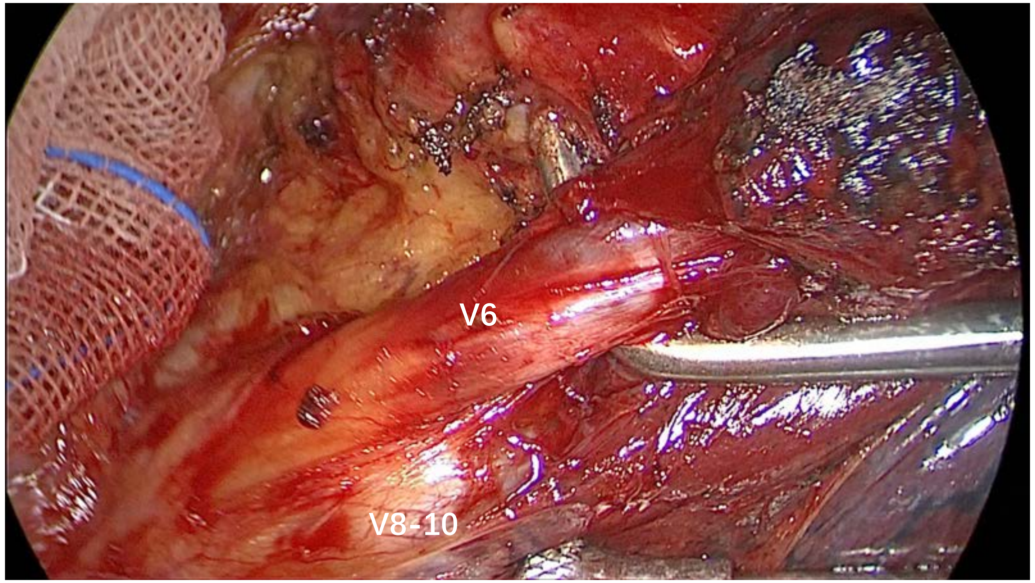

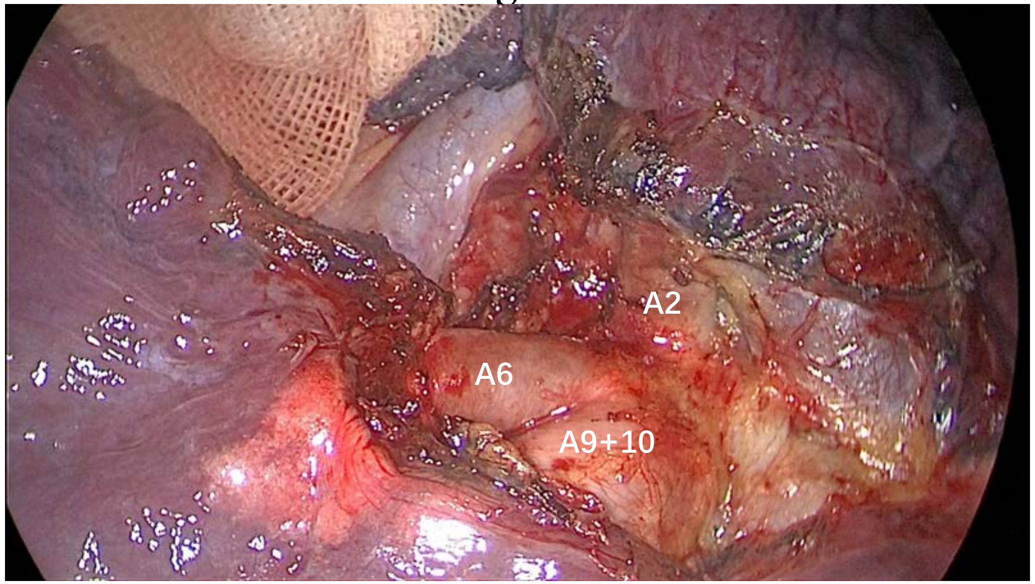

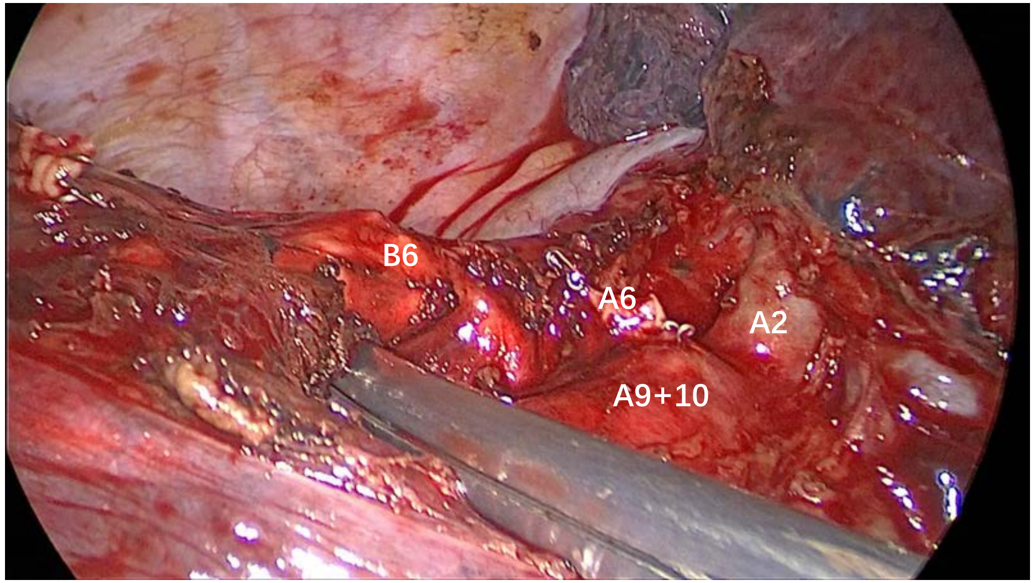

Supplement: Supplementary file 1 — Figure S1 The intraoperative observation of enrolled 20 patients and the 3‐D reconstruction by AI and manual approach. [file TCA-13-795-s002.pdf]
