# Supplementary material for: A fully automated noncontrast CT 3‐D reconstruction algorithm enabled accurate anatomical demonstration for lung segmentectomy
Source: Thorac Cancer. 2022 Feb 9;13(6):795–803. doi: 10.1111/1759-7714.14322 (PMC8930461; doi:10.1111/1759-7714.14322)
Supplement: Supplementary file 2 — Table S1 Supplementary Tables. [file TCA-13-795-s001.zip › TCA_14322_Supplementary table 5.docx]

| **Table S4** Error case of AI reconstruction during the independent performance assessment | | | |
| --- | --- | --- | --- |
| Patient id | Type | Structure | Lobe |
| 3 | Misclassification | Vein | RLL |
| 4 | Both | Vein | RUL |
| 5 | Misdetection | Vein | RUL |
| 8 | Misdetection | Vein | LLL |
| 11 | Misclassification | Vein | RUL |
| 13 | Misclassification | Artery | RUL |
