# Supplementary material for: A fully automated noncontrast CT 3‐D reconstruction algorithm enabled accurate anatomical demonstration for lung segmentectomy
Source: Thorac Cancer. 2022 Feb 9;13(6):795–803. doi: 10.1111/1759-7714.14322 (PMC8930461; doi:10.1111/1759-7714.14322)
Supplement: Supplementary file 2 — Table S1 Supplementary Tables. [file TCA-13-795-s001.zip › TCA_14322_Supplementary table.pdf]

Table S1A Anatomical variants of right upper lobe.

Right Upper Lobe	Artery				Vein				Bronchus	
	Superior pulmonary artery	A1	A2	A3	Superior pulmonary vein	V1	V2	V3	Upper lobe bronchus	
Type 1	No inter-lobular variant	Both A1a and A1b from truncus superior artery	A2a from recurrent artery; A2b from ascending artery	Both A3a and A3b from truncus superior artery	No inter-lobular variant	Both V1 and V2 veins	Both V1 and V2 veins	Both V3a and V3b	No inter-lobular variant	Three branches into B1, B2, and B3
Type 2	A2 from A6	A1a branches independently, A1b from truncus superior artery	Both A2a and A2b from ascending artery	A3a from truncus intermedius; A3b from truncus superior artery	V2 running into inferior pulmonary vein	Apical vein without central vein: V1 and V2 form common branch, running ventral to the hilum. V2 branches along this route, running into the lung and between ascending and truncus superior arteries.	V2 from V1	V3a and V3b form a common trunk	Upper lobe bronchus from main bronchus	Two branches into B1+3 and B2
Type 3	Other	Other	Both A2a and A2b from recurrent artery	A3a from truncus superior; A3b from truncus intermedius	Middle lobe vein running into a high site	V1 from V2	Central vein without apical vein: common branch comprising V1 and V2 runs as central vein. V1 branches along this route running cranially inside the lung.	Other	Other	B1+2 and B3
Type 4			Other	Other	V2 and V6 form a common trunk running into left atrium	Other	Other			B1+B2+3
Type 5					V2 running into left atrium independently					Four branches into others
Type 6					Other					Other

Table S1B Anatomical variants of right middle lobe.

Right Middle Lobe	Artery		Vein		Bronchus	
Type 1	A4 and A5 share a same truncus	Branches from main pulmonary artery	V4 and V5 forms a common truncus	V4+5 running into central vein at the site lower than horizontal fissure	B4 and B5 forms a common truncus	From truncus intermedius bronchus
Type 2	A4 and A5 are independent	A4 branches from basal artery, A5 branches from main pulmonary artery	V4 and V5 are independent	V4+5 running into central vein at the site higher than horizontal fissure	B4 and B5 are independent	From upper lobar bronchus
Type 3	Other	A5 branches from basal artery, A4 branches from main pulmonary artery	Other	V4+5 running into inferior pulmonary vein	Three branches	Other
Type 4		Other		V4+5 running into superior pulmonary vein	Other	
Type 5				Other		

Table S1C Anatomical variants of right lower lobe.

Right Lower Lobe	Artery							Vein					Bronchus						
	Inferior pulmonary artery		A6	A7	A8	A9	A10	Inferior pulmonary vein	V6	V8	v9	V10	Lower lobe bronchus		B6	B7	B8	B9	B10
Type 1	A*	No inter-lobular variant	One branch	Forms common truncus with A8	Forms common truncus with A7	Forms common truncus with A10	Forms common truncus with A9	No inter-lobular variant	V6a, V6b, V6c	Forms common truncus with part of V9	Part of V9 forms common truncus with V8 and the rest of V9 forms common truncus with V10	Part of V9 forms common truncus with V10	B*	No inter-lobular variant	B6a+c and B6b	Both B7a and B7b run ventral to inferior pulmonary vein	Forms common truncus with B9	Forms common truncus with B10	Forms common truncus with B9
Type 2	Absent A*	A6 from A2	Two branches	Branches from basal pulmonary artery	Forms common truncus with A9	Forms common truncus with A8	A10	V6 runs into superior pulmonary vein	V6a+b、V6c	Forms common truncus with V9 and part of V10	Part of V10 forms common truncus with V8 and V9	Part of V10 independent, the rest of V10 forms common truncus with V9 and V8	Absent B*	Other	B6a+b and B6c	B7a and B7b straddle inferior pulmonary vein	B8 and B9 and B10	Forms common truncus with B8	B8 and B9 and B10
Type 3		A7 from middle lobe artery	Three branches	Absent A7	Independent	Independent	Independent	Other	V6a+c、V6b	Part of V8 independent, the rest of V8 forms common truncus with V9 and V10	Part of V8 forms common truncus with V9 and V10	Part of V8 forms common truncus with V9 and V10			B6a and B6b and B6c	Lack of B7	Other	B8 and B9 and B10	Other
Type 4		Other	Other	Independent	Other	Other	Other		V6b+c、V6a	Forms common truncus with V9	Forms common truncus with V8	Independent			Other	Other		Other	
Type 5				Other					V6a+b+c	Independent	Forms common truncus with V10	Forms common truncus with V9							
Type 6										Other	Independent	Independent							
Type 7											Other	Other							

Table S1D Anatomical variants of left upper lobe.

Left Upper Lobe	Artery						Vein			Bronchus					
	Superior pulmonary artery	A1+2	A1+2d	A3	A4	A5	Superior pulmonary vein	V1+2+3	V4+5	Upper lobe bronchus	B1-3	B1+2	B3	B4	B5
Type 1	No inter-lobular variant	A1+2a+b and A1+2c	Absent	A3 branches from main pulmonary artery	A4 branch from interlobar artery	A5 branch from interlobar artery	No inter-lobular variant	Apical type: V1+2 and V3 both branch from superior pulmonary vein	Forms common truncus	No inter-lobular variant	B1+2 and B3	B1+2a+b and B1+2c	B3a and B3b+c	Forms common truncus with B5	Forms common truncus with B4
Type 2	A2 from A6	A1+2a, A1+2b, and A1+2c	One	A3b+c branches from main pulmonary artery, and A3a branches distal to A1+2c	A4+5 branches from interlobar artery	A4+5 branches from interlobar artery	Lingual vein running into inferior pulmonary vein	Central type: entire V1+2 forms a common truncus with V3, and runs into deep lung tissue	Other	Lingual bronchus branches from lower lobe	B1+2, B3a and B3b+c	B1+2a and B1+2b+c	Other	Other	Other
Type 3	A4+5 from basal artery	A1+2a and A1+2b+c	Other	Other	A4+5 branches from mediastinum (main pulmonary artery) and runs between V1+3 and B3	A4+5 branches from mediastinum (main pulmonary artery) and runs between V1+3 and B3	Other	Other		Other	B1+2a+b, B1+2c, and B3	Other			
Type 4	Other	A1+2a+b+c			A4 branches from mediastinum (main pulmonary artery) and runs between V1+3 and B3	A5 branches from mediastinum (main pulmonary artery) and runs between V1+3 and B3					Other				
Type 5		Other			Other	Other									

Table S1E Anatomical variants of left lower lobe.

Left Lower Lobe	Artery						Vein					Bronchus					
	Inferior pulmonary artery		A6	A8	A9	A10	Inferior pulmonary vein	V6	V8	v9	V10	Lower lobe bronchus		B6	B8	B9	B10
Type 1	A*	No inter-lobular variant	One branch	Independent	Forms common truncus with A10	Forms common truncus with A9	No inter-lobular variant	V6a, V6b, V6c	Forms common truncus with part of V9	Part of V9 forms common truncus with V8 and the rest of V9 forms common truncus with V10	Part of V9 forms common truncus with V10	B*	No inter-lobular variant	B6a+c and B6b	Independent	Forms common truncus with B10	Forms common truncus with B9
Type 2	Absent A*	A6 from A2	Two branches	Forms common truncus with A9	Forms common truncus with A8	Independent	V6 runs into superior pulmonary vein	V6a+b、V6c	Forms common truncus with V9 and part of V10	Part of V10 forms common truncus with V8 and V9	Part of V10 independent, the rest of V10 forms common truncus with V9 and V8	Absent B*	Other	B6a+b and B6c	Forms common truncus with B9	Forms common truncus with B8	Independent
Type 3		Other	Other	Other	Independent	Other	Lingual vein running into inferior pulmonary vein	V6a+c、V6b	Part of V8 independent	Part of V8 forms common truncus with V9 and V10	Part of V8 forms common truncus with V9 and V10			B6a and B6b and B6c	Other	Independent	Other
Type 4					Other		Other	V6b+c、V6a	Forms common truncus with V9	Forms common truncus with V8	Forms common truncus with V9			B6a and B6b+c		Other	
Type 5								V6a+b+c	Independent	Forms common truncus with V10	Independent			Other			
Type 6									Other	Independent	Other						
Type 7										Other							

Table S2 A scoring system for the assessment of quality of CT image.

**Score**	**Overall assessment**	**Breathing or motion artifacts**
5	Excellent quality	No visible breathing or motion artifact
4	Good quality	Slightly visible breathing or motion artifacts, but all relevant bronchial and vascular bifurcations can be identified
3	General quality	A small number of visible breathing or motion artifacts, but general relevant bronchial and vascular bifurcations can be identified
2	Poor quality	Visible breathing or motion artifacts obscure several bronchial and vascular bifurcations
1	Extremely poor quality, rejected	A large number of visible breathing or motion artifacts obscure all relevant bronchial and vascular bifurcations

Table S3 Detailed dicom information of patients.

	Sex	Age	Date	Time	StudyInstanceUID	SeriesInstanceUID	KVP	XRayTubeCurrent	Manufacturer	ManufacturerModelName	PixelSpacing	BodyPartExamined	ConvolutionKernel	WindowWidth	WindowCenter	SliceThickness	Rows	Columns	SeriesDescription
1	F	037Y	20200430	151829	1.2.840.113619.2.416.132681632156592044263730001262515892032	1.2.840.113619.2.416.205015962559820965132797817375419829912	"100.0"	141	GE MEDICAL SYSTEMS	Revolution CT	[0.669922, 0.669922]	1.25 mm stnd	STANDARD	40	350	"1.25"	512	512	1.25 mm stnd
2	F	066Y	20200508	153326	1.2.840.113619.2.416.207437618084707403934862838113165538522	1.2.840.113619.2.416.44291536488251030194529107838054826388	"100.0"	393	GE MEDICAL SYSTEMS	Revolution CT	[0.771484, 0.771484]	1.25 mm stnd	STANDARD	40	350	"1.25"	512	512	1.25 mm stnd
3	F	053Y	20200520	164823	1.2.840.113619.186.80861568102.20200520164331187.702	1.2.840.113619.2.55.3.1124731650.639.1589929700.891.4	"120.0"	360	GE MEDICAL SYSTE	LightSpeed VCT	[0.638672, 0.638672]	Recon 3:	STANDARD	40	400	"1.25"	512	512	Recon 3:

									MS										
4	F	062Y	20200531	74742	1.2.840.113619.186.80861568102.20200531074340703.230	1.2.156.112605.189250940724437.200530234742.3.3404.67205	"120.0"	120	UIH	uCT 760	[0.68359375, 0.68359375]	1mm soft B	B_SOFT_B	40	400	"1.0"	512	512	1mm soft B
5	F	053Y	20200610	83423	1.2.840.113619.186.80861568102.20200610083314460.350	1.2.156.112605.189250940724437.200610003422.3.3404.66323	"120.0"	161	UIH	uCT 760	[0.68359375, 0.68359375]	1mm soft B	B_SOFT_B	40	400	"1.0"	512	512	1mm soft B
6	F	059Y	20200611	184007.473	1.2.840.113619.186.80861568102.20200611175300362.744	1.3.12.2.1107.5.1.4.76134.30000020061023530263000175424	"100.0"	98	SIEMENS	SOMATOM Force	[0.734375, 0.734375]	Thorax 1.0 Br40 3	['Br40d', '3']	[55, -600]	[360, 1200]	"1.0"	512	512	Thorax 1.0 Br40 3
7	F	040Y	20200715	173611.538	1.2.840.113619.186.80861568102.20200715173144595.115	1.3.12.2.1107.5.1.4.76134.30000020071423580063500182312	"110.0"	262	SIEMENS	SOMATOM Force	[0.7578125, 0.75781	Thorax 1.0 Br40 3	['Br40d', '3']	[50, -600]	[350, 1200]	"1.0"	512	512	Thorax 1.0 Br40 3

											25]								
8	M	064Y	20200722	142830	1.2.840.113619.186.80861568102.20200722141358451.913.111111.100018	1.2.840.113619.2.55.3.1124731650.746.1595392953.183.4.111111.100018	"120.0"	N/A	GE MEDICAL SYSTEMS	LightSpeed VCT	[0.808594, 0.808594]	Recon 3:	STANDARD	40	400	"1.25"	512	512	Recon 3:
9	F	065Y	20200724	112350.598	1.2.840.113619.186.80861568102.20200724111237539.663	1.3.12.2.1107.5.1.4.76134.30000020072323530453900094310	"100.0"	144	SIEMENS	SOMATOM Force	[0.693359375, 0.693359375]	Thorax 1.0 Br40 3	['Br40d', '3']	[55, -600]	[360, 1200]	"1.0"	512	512	Thorax 1.0 Br40 3
10	M	058Y	20200730	140456	1.2.840.113619.186.80861568102.20200730140330046.234.111111.100000	1.2.840.113619.2.55.3.1124731650.748.1595836996.837.4.111111.100000	"120.0"	360	GE MEDICAL SYSTEMS	LightSpeed VCT	[0.703125, 0.703125]	Recon 3:	STANDARD	40	400	"1.25"	512	512	Recon 3:
11	F	050	20200808	75921	1.2.840.113619.186.80861568102.20200817080235126.329	1.2.156.112605.189250946104227.200816235920.3.5100.63250	"120.	123	UIH	uCT 780	[0.6835937	1mm soft B	B_SOFT_B	40	400	"1.0"	512	512	1mm soft B

		Y	17				0"				5, 0.68359375]								
12	F	041Y	20200609	122803	1.2.840.113619.186.80861568102.20200609122504317.590	1.2.156.112605.189250940724437.200609042803.3.3404.74005	"120.0"	141	UIH	uCT 760	[0.68359375, 0.68359375]	1mm soft B	B_SOFT_B	40	400	"1.0"	512	512	1mm soft B
13	M	063Y	20200721	181207.135	1.2.840.113619.186.80861568102.20200721180743424.708	1.3.12.2.1107.5.1.4.76134.30000020072023430205300188732	"100.0"	143	SIEMENS	SOMATOM Force	[0.658203125, 0.658203125]	Thorax 1.0 Br40 3	['Br40d', '3']	[55, -600]	[360, 1200]	"1.0"	512	512	Thorax 1.0 Br40 3
14	M	059Y	20200722	75204.969	1.2.840.113619.186.80861568102.20200722072247820.591	1.3.12.2.1107.5.1.4.76134.30000020072123414878700005605	"110.0"	44	SIEMENS	SOMATOM Force	[0.751953125, 0.75195312	Thorax 1.0 Br40 3	['Br40d', '3']	[50, -600]	[350, 1200]	"1.0"	512	512	Thorax 1.0 Br40 3

											5]							
15	F	062Y	20200813	82209	1.2.840.113619.186.80861568102.20200813082320749.163	1.2.156.112605.189250940724437.200813002209.3.6224.67859	"120.0"	224	UIH	uCT 760	[0.68359375, 0.68359375]	1mm soft B	B_SOFT_B	40	400	"1.0"	512	512	1mm soft B
16	F	049Y	20200712	151538.113	1.2.840.113619.186.80861568102.20200712150432811.714	1.3.12.2.1107.5.1.4.76134.30000020070923542822000352753	"100.0"	109	SIEMENS	SOMATOM Force	[0.673828125, 0.673828125]	Thorax 1.0 Br40 3	['Br40d', '3']	[55, -600]	[360, 1200]	"1.0"	512	512	Thorax 1.0 Br40 3
17	F	058Y	20200724	134324.402	1.2.840.113619.186.80861568102.20200724134025282.630	1.3.12.2.1107.5.1.4.76134.30000020072323530453900126555	"100.0"	238	SIEMENS	SOMATOM Force	[0.724609375, 0.724609375]	Thorax 1.0 Br40 3	['Br40d', '3']	[55, -600]	[360, 1200]	"1.0"	512	512	Thorax 1.0 Br40 3
18	F	05	2020	1602	1.2.840.113619.186.80861568102.202004021	1.3.12.2.1107.5.1.4.76134.3000002004012358	"10	417	SIEME	SOMATOM	[0.7148	Thorax	['Br40d',	[55, -	[360 ,	"1.0"	51	512	Thorax

		1Y	0402	08.052	55914239.942	0872100102072	0.0"		NS	Force	4375, 0.71484375]	1.0 Br403	'3']	600]	1200]		2		1.0 Br403
19	M	063Y	20200805	92706	1.2.840.113619.186.80861568102.20200805092552741.669	1.2.156.112605.189250940724437.200805012706.3.6224.66496	"120.0"	120	UIH	uCT 760	[0.68359375, 0.68359375]	1mm soft B	B_SOFT_B	40	400	"1.0"	512	512	1mm soft B
20	M	056Y	20200528	193317.502	1.2.840.113619.186.80861568102.20200528184420568.447	1.3.12.2.1107.5.1.4.76134.30000020052800043791200156479	"100.0"	238	SIEMENS	SOMATOM Force	[0.751953125, 0.751953125]	Thorax 1.0 Br403	['Br40d', '3']	[55, -600]	[360, 1200]	"1.0"	512	512	Thorax 1.0 Br403

Table S4A Real-world performance assessment for all variance.

			Gold standard		Agent A	Agent B	Agent C	Agent D
	Question	Type	Surgery	Variance	Variance	Variance	Variance	Variance
Case 1	A1+2	A	LS1+2+3	2	2	2	2	2
	A3	A		3	3	3	3	3
	V1+2+3	V		1	1	1	1	1
	B1-3	B		3	3	3	3	3
	B1+2	B		1	1	1	1	1
	B3	B		1	1	1	1	1
Case 2	A10	A		1	2	2	2	2
	V10	V		6	4	4	1	1
	B10	B		3	NA	2	2	3
Case 3	A1	A	RS1	1	1	1	1	1
	V1	V		1	1	1	1	1
	B1-3	B		1	1	1	1	1
Case 4	A6	A	RS6	1	1	2	2	1
	V6	V		5	5	5	4	4
	B6	B		1	1	1	1	1
Case 5	A4	A	LS4+5	1	1	1	1	1
	A5	A		3	3	3	3	3
	V4+5	V		2	1	1	2	2
	B4	B		1	1	1	1	1
	B5	B		1	1	1	1	1
Case 6	A2	A	RS2	2	2	2	2	2
	V2	V		3	3	3	3	3
	B1-3	B		1	1	1	1	1
Case 7	A2	A	RS2	1	1	1	1	1
	V2	V		3	3	3	4	3
	B1-3	B		5	5	5	5	5
Case 8	A2	A	RS2+3	2	2	2	2	NA
	A3	A		1	1	1	1	1
	V2	V		1	1	1	1	1
	V3	V		2	2	2	2	2
	B1-3	B		1	1	1	1	1
Case 9	A1+2	A	LS1+2+3	1	1	1	1	1
	A3	A		1	3	NA	3	1
	V1+2+3	V		1	1	1	1	1
	B1-3	B		1	1	NA	1	1
	B1+2	B		1	1	1	1	1
Case 10	A6	A	RS6	1	1	1	1	1
	V6	V		2	2	2	2	2
	B6	B		1	1	1	1	1
Case 11	A6	A	LS6	2	2	2	2	2
	V6	V		1	5	1	5	5
	B6	B		2	2	2	3	2
Case 12	A9	A	LS9+10	1	1	1	1	1
	A10	A		1	1	1	1	1
	V9	V		1	1	1	1	1
	V10	V		1	1	1	1	2
	B9	B		1	1	1	1	1
	B10	B		1	1	1	1	1
Case 13	A4	A	LS4+5	1	1	1	1	1
	A5	A		1	1	1	1	1
	V4+5	V		1	1	1	1	1
	B4	B		1	1	1	1	1
	B5	B		1	1	1	1	1
Case 14	A9	A		1	1	1	1	1
	V9	V		1	NA	NA	1	1
	B9	B		1	1	1	1	1
Case 15	A9	A	LS9+10	3	3	1	1	1
	A10	A		2	2	1	1	1
	V9	V		6	6	6	6	6
	V10	V		4	4	4	4	4
	B9	B		1	1	1	1	1
	B10	B		1	1	1	1	1
Case 16	A8	A	RS8	3	4	3	3	4
	V8	V		5	5	5	5	4
	B8	B		2	2	2	2	2
Case 17	A3	A	RS3	4	1	1	2	2
	V3	V		1	1	1	2	3
	B1-3	B		1	1	1	1	1
Case 18	A2	A	RS2	1	1	1	1	1
	V2	V		1	1	1	1	1
	B1-3	B		1	1	1	1	1
Case 19	A9	A	LS9+10	1	1	1	1	1
	A10	A		1	1	1	1	1
	V9	V		6	4	6	6	6
	V10	V		4	4	4	4	4
	B9	B		4	4	4	4	4
	B10	B		3	3	3	3	3
Case 20	A8	A	LS8	1	1	1	1	2
	V8	V		5	5	5	5	5
	B8	B		1	1	1	1	1
					Agent A	Agent B	Agent C	Agent D
Accuracy			Average		Variant	Variant	Variant	Variant
All variance			0.85		0.88	0.86	0.84	0.84

Table S4B Real-world performance assessment for pulmonary arteries.

			Gold standard		Agent A	Agent B	Agent C	Agent D
	Question	Type	Surgery	Variance	Variance	Variance	Variance	Variance
Case 1	A1+2	A	LS1+2+3	2	2	2	2	2
	A3	A		3	3	3	3	3
Case 2	A10	A		1	2	2	2	2
Case 3	A1	A	RS1	1	1	1	1	1
Case 4	A6	A	RS6	1	1	2	2	1
Case 5	A4	A	LS4+5	1	1	1	1	1
	A5	A		3	3	3	3	3
Case 6	A2	A	RS2	2	2	2	2	2
Case 7	A2	A	RS2	1	1	1	1	1
Case 8	A2	A	RS2+3	2	2	2	2	NA
	A3	A		1	1	1	1	1
Case 9	A1+2	A	LS1+2+3	1	1	1	1	1
	A3	A		1	3	NA	3	1
Case 10	A6	A	RS6	1	1	1	1	1
Case 11	A6	A	LS6	2	2	2	2	2
Case 12	A9	A	LS9+10	1	1	1	1	1
	A10	A		1	1	1	1	1
Case 13	A4	A	LS4+5	1	1	1	1	1
	A5	A		1	1	1	1	1
Case 14	A9	A		1	1	1	1	1
Case 15	A9	A	LS9+10	3	3	1	1	1
	A10	A		2	2	1	1	1
Case 16	A8	A	RS8	3	4	3	3	4
Case 17	A3	A	RS3	4	1	1	2	2
Case 18	A2	A	RS2	1	1	1	1	1
Case 19	A9	A	LS9+10	1	1	1	1	1
	A10	A		1	1	1	1	1
Case 20	A8	A	LS8	1	1	1	1	2
					Agent A	Agent B	Agent C	Agent D
Accuracy			Average		Variant	Variant	Variant	Variant
PAs			0.79		0.86	0.79	0.79	0.75

Table S4C Real-world performance assessment for pulmonary veins.

			Gold standard		Agent A	Agent B	Agent C	Agent D
	**Question**	**Type**	**Surgery**	**Variance**	**Variance**	**Variance**	**Variance**	**Variance**
Case 1	V1+2+3	V		1	1	1	1	1
Case 2	V10	V		6	4	4	1	1
Case 3	V1	V		1	1	1	1	1
Case 4	V6	V		5	5	5	4	4
Case 5	V4+5	V		2	1	1	2	2
Case 6	V2	V		3	3	3	3	3
Case 7	V2	V		3	3	3	4	3
Case 8	V2	V		1	1	1	1	1
Case 8	V3	V		2	2	2	2	2
Case 9	V1+2+3	V		1	1	1	1	1
Case 10	V6	V		2	2	2	2	2
Case 11	V6	V		1	5	1	5	5
Case 12	V9	V		1	1	1	1	1
Case 12	V10	V		1	1	1	1	2
Case 13	V4+5	V		1	1	1	1	1
Case 14	V9	V		1	NA	NA	1	1
Case 15	V9	V		6	6	6	6	6
Case 15	V10	V		4	4	4	4	4
Case 16	V8	V		5	5	5	5	4
Case 17	V3	V		1	1	1	2	3
Case 18	V2	V		1	1	1	1	1
Case 19	V9	V		6	4	6	6	6
Case 19	V10	V		4	4	4	4	4
Case 20	V8	V		5	5	5	5	5
					**Agent A**	**Agent B**	**Agent C**	**Agent D**
**Accuracy**			**Average**		**Variant**	**Variant**	**Variant**	**Variant**
PVs			0.80		0.79	0.88	0.79	0.75

Table S4D Real-world performance assessment for bronchi.

			Gold standard		Agent A	Agent B	Agent C	Agent D
	Question	Type	Surgery	Variance	Variance	Variance	Variance	Variance
Case 1	B1-3	B		3	3	3	3	3
	B1+2	B		1	1	1	1	1
	B3	B		1	1	1	1	1
Case 2	B10	B		3	NA	2	2	3
Case 3	B1-3	B		1	1	1	1	1
Case 4	B6	B		1	1	1	1	1
Case 5	B4	B		1	1	1	1	1
	B5	B		1	1	1	1	1
Case 6	B1-3	B		1	1	1	1	1
Case 7	B1-3	B		5	5	5	5	5
Case 8	B1-3	B		1	1	1	1	1
Case 9	B1-3	B		1	1	NA	1	1
	B1+2	B		1	1	1	1	1
Case 10	B6	B		1	1	1	1	1
Case 11	B6	B		2	2	2	3	2
Case 12	B9	B		1	1	1	1	1
	B10	B		1	1	1	1	1
Case 13	B4	B		1	1	1	1	1
	B5	B		1	1	1	1	1
Case 14	B9	B		1	1	1	1	1
Case 15	B9	B		1	1	1	1	1
	B10	B		1	1	1	1	1
Case 16	B8	B		2	2	2	2	2
Case 17	B1-3	B		1	1	1	1	1
Case 18	B1-3	B		1	1	1	1	1
Case 19	B9	B		4	4	4	4	4
	B10	B		3	3	3	3	3
Case 20	B8	B		1	1	1	1	1
					Agent A	Agent B	Agent C	Agent D
Accuracy			Average		Variant	Variant	Variant	Variant
Bronchi			0.96		0.96	0.93	0.93	1.00
